# Differential Contribution of P5CS Isoforms to Stress Tolerance in Arabidopsis

**DOI:** 10.3389/fpls.2020.565134

**Published:** 2020-09-25

**Authors:** Dietmar Funck, Lukas Baumgarten, Marc Stift, Nicolaus von Wirén, Luise Schönemann

**Affiliations:** ^1^ Department of Biology, University of Konstanz, Konstanz, Germany; ^2^ Molecular Plant Nutrition, Leibniz Institute of Plant Genetics and Crop Plant Research, Gatersleben, Germany

**Keywords:** proline biosynthesis, pyrroline-5-carboxylate synthetase, salt stress, compatible solutes, subcellular localization, seedling development, *Pseudomonas syringae*

## Abstract

Proline accumulation is a widespread response of plants to salt stress as well as drought and cold stress. In most plant species, two isoforms of pyrroline-5-carboxylate synthetase (P5CS) catalyze the first step in proline biosynthesis from glutamate. In Arabidopsis, these isoforms differ in their spatial and temporal expression patterns, suggesting sub-functionalization. P5CS1 has been identified as the major contributor to stress-induced proline accumulation, whereas P5CS2 has been considered important for embryo development and growth. In contrast to previous results, our analysis of P5CS1- and P5CS2-GFP fusion proteins indicates that both enzymes were exclusively localized in the cytosol. The comparison of the susceptibility of *p5cs1* and *p5cs2* mutants to infection with *Pseudomonas syringae* and salt stress provided novel information on the contribution of the two P5CS isoforms to proline accumulation and stress tolerance. In agreement with previous studies, salt-stressed *p5cs1* mutants accumulated very little proline, indicating that P5CS1 contributed more to stress-induced proline accumulation, whereas its impact on stress tolerance was rather weak. Germination and establishment of *p5cs2* mutants were impaired under ambient conditions, further supporting that P5CS2 is most important for growth and development, whereas its contribution to stress-induced proline accumulation was smaller than that of P5CS1. In contrast to *p5cs1* mutants or wildtype plants, *p5cs2* mutants were only weakly affected by sudden exposure to a high NaCl concentration. These findings show that proline content, which was intermediate in leaves of *p5cs2* mutants, was not directly correlated with stress tolerance in our experiments. In rosettes of NaCl-exposed *p5cs2* mutants, nearly no accumulation of Na^+^ was observed, and the plants showed neither chlorosis nor reduction of photosynthesis. Based on these data, we suggest a function of P5CS2 or P5CS2-mediated proline synthesis in regulating Na^+^ accumulation in leaves and thereby salt stress tolerance.

## Introduction

Worldwide, soil salinity is a major problem for plant productivity in irrigation-dependent cultivation systems as well as in coastal areas. A high soluble ion content in the soil constitutes two problems for plant growth: First, water uptake is inhibited due to its dependence on a higher concentration of osmotically active solutes inside the cells. Second, some ions, especially Na^+^, exert toxic effects when they accumulate inside cells (Munns and Tester, 2008; Zörb et al., 2019). Since Na^+^ and Cl^−^ are usually the most abundant ions in saline soil, most experimental studies use NaCl-induced stress as a proxy for salt stress in general. Numerous efforts have been made to improve the salt tolerance of crop plants while at the same time maintaining high productivity under non-stress conditions (Agarwal et al., 2013; Liang et al., 2018). However, the progress achieved so far is rather limited, emphasizing the need for a more detailed analysis of the natural tolerance mechanisms that plants have evolved to cope with salt stress.

Arabidopsis and many other plant species accumulate free proline in response to salt stress or similar environmental stress factors causing cellular water deprivation (Liang et al., 2013; Kavi Kishor and Sreenivasulu, 2014). Stress-dependent accumulation of proline is achieved by induced biosynthesis from glutamate in combination with proline transport between different tissues or cell types as well as repression of proline degradation in mitochondria (Szabados and Savoure, 2010; Trovato et al., 2019). Stress-induced alterations of protein biosynthesis and degradation rates may also affect free amino acid concentrations, but are not expected to cause a specific increase in proline (Hildebrandt, 2018). High concentrations of proline can stabilize proteins and membranes, and it has been suggested that proline accumulation stimulates the antioxidant defense system (Forlani et al., 2019b; Zouari et al., 2019). Direct contributions of proline to radical scavenging and osmotic adjustment have also been proposed but the evidence is controversial (Forlani et al., 2019a). At present, it is not fully understood whether high proline concentrations or the synthesis of proline mediate the protective effects.

Proline biosynthesis is a two-step process, in which the intermediate pyrroline-5-carboxylate (P5C) is synthesized by the bifunctional enzyme P5C-synthetase (P5CS), combining both glutamate kinase and γ-glutamylphosphate reductase activities in a single polypeptide. Like many other plant species, Arabidopsis has two isoforms of P5CS, which apparently are sub-functionalized in the stress-responsive P5CS1 (At2g39800) and the housekeeping P5CS2 (At3g55610; (Székely et al., 2008; Turchetto-Zolet et al., 2009). Analysis of GFP fusion proteins suggested that both P5CS isoforms are mainly cytosolic but may be localized in plastids under stress conditions (Székely et al., 2008). The second step of proline biosynthesis is catalyzed by P5C reductase (P5CR), which is encoded by a single-copy gene in Arabidopsis and most other plant species. The expression pattern of *P5CR* did not provide conclusive evidence for a limiting role in proline biosynthesis, giving rise to the assumption that P5CS is the key enzyme for proline accumulation (Szabados and Savoure, 2010). However, the detailed characterization of post-translational mechanisms of enzyme activity modulation provided evidence that also P5CR activity may be regulating proline synthesis rates according to the cellular redox status and cytosolic ion concentrations (Giberti et al., 2014). A P5CR:GFP fusion protein was exclusively localized in the cytosol in Arabidopsis, raising the question whether plastids can indeed be the site of proline synthesis in stressed plants (Funck et al., 2012).

Several independent T-DNA insertion mutants lacking P5CS1 have shown strongly reduced proline levels both under normal growth conditions and in response to stress. This had been revealed by increased levels of reactive oxygen species in leaves of stressed *p5cs1* mutants, and by root growth being more sensitive to NaCl- or PEG-induced osmotic stress compared to wildtype plants (Székely et al., 2008; Sharma et al., 2011). Expression of P5CR and at least one isoform of P5CS are essential for sexual reproduction in Arabidopsis, indicating that plants have no alternative pathways to synthesize proline (Funck et al., 2012; Mattioli et al., 2012; Mattioli et al., 2018). Homozygous *p5cs1* mutants that were additionally heterozygous for a *p5cs2* mutation had very low proline content and root growth was reduced due to fewer cell divisions in the meristematic zone of the root tip (Biancucci et al., 2015). In contrast to single mutants lacking P5CS1, which develop normally and are fertile, homozygous embryos of two recessive T-DNA insertion mutants lacking P5CS2 are conditionally aborted in heterozygous plants (Székely et al., 2008; Mattioli et al., 2009; Funck et al., 2012). When immature, homozygous *p5cs2* seeds were fed with proline, they germinated *in vitro* and the resulting seedlings were shown to be devoid of functional *P5CS2* transcripts (Székely et al., 2008). We succeeded to cultivate rescued, homozygous *p5cs2* seedlings on soil, where they produced viable seeds under short-day conditions (Funck et al., 2012). The stress tolerance of *p5cs2* mutants has not been analyzed so far.

Besides salt and osmotic stress, also biotic stress leads to alterations in proline metabolism. *P5CS1* expression did not change considerably in response to bacterial infections, whereas an upregulation of *P5CS2* during the hypersensitive response to an avirulent *Pseudomonas syringae* strain was observed in Arabidopsis (Fabro et al., 2004). Multiple lines of evidence suggest a role of proline degradation in the orchestration of the hypersensitive response to incompatible pathogens (Ayliffe et al., 2002; Fabro et al., 2004; Mitchell et al., 2006; Cecchini et al., 2011). Especially proline dehydrogenase (ProDH)-dependent generation of reactive oxygen species in mitochondria seems to contribute to the regulation of cell death or survival, but also signaling functions or toxicity of mitochondrial P5C, or a proline-P5C cycle across the mitochondrial membrane were proposed (Deuschle et al., 2004; Miller et al., 2009; Cecchini et al., 2011; Monteoliva et al., 2014). The individual contributions of P5CS1 and P5CS2 to pathogen defense have not yet been reported.

In this study, we revisited the subcellular localization of the two P5CS isoforms in Arabidopsis and used *p5cs1* and *p5cs2* mutants to perform a detailed analysis of their importance for germination and seedling development. Additionally, we tested the pathogen susceptibility of mature plants and analyzed their sensitivity to salt and excess light stress alone or in combination. Our results indicate that *P5CS2* expression does not contribute to the defense against *P. syringae* but has a regulatory function for NaCl stress tolerance.

## Materials and Methods

### Plant Material and DNA Constructs

Arabidopsis (*Arabidopsis thaliana* (L.) Heynh., ecotype Col-0, seed stock Col-8 from NASC) and T-DNA insertion lines were obtained from the NASC (GABI452_G01, *p5cs2-1*; Salk_063517, *p5cs1-4*) or from the INRA Versailles Resource Centre (FLAG_139H07, *p5cs2-2*). Backcrossing of the mutants and generation of homozygous plants is described in (Funck et al., 2012). Expression constructs for P5CS:GFP fusion proteins were generated by inserting the coding sequences without stop codon of *P5CS1* (from ABRC clone U14433) or *P5CS2* (from ABRC clone G2E1) *via* pENTR into the plant transformation vector pUBC-GFP-Dest (Grefen et al., 2010) and verified by Sanger sequencing. *Agrobacterium tumefaciens* strain GV3101 was used to transform Col-0 plants by the floral dip method (Clough and Bent, 1998).

### Confocal Microscopy

Protoplasts were isolated from leaves of P5CS1:GFP or P5CS2:GFP expressing and wildtype plants by overnight incubation in protoplast medium (0.45 M sorbitol, ½ strength MS salt mixture) supplemented with 10 mg ml^−1^ cellulase and 2.5 mg ml^−1^ macerozyme. Spectral images were recorded with a Zeiss LSM880 equipped with a 63× water immersion lens. The samples were excited at 488 nm and images were recorded with 20 spectral channels (9 nm bandwidth) between 490 and 668 nm. A chlorophyll spectrum was obtained from WT protoplasts and a GFP spectrum from a P5CS2:GFP expressing epidermis protoplast that did not contain chloroplasts. Linear unmixing with background subtraction was performed with the ZEN software (Zeiss). Channel overlay, false coloring and adjustment to identical gain, offset and contrast settings were performed in ImageJ and Adobe Photoshop.

### Growth Conditions and Stress Treatments

For germination assays, the assessment of seedling development or root growth and RNA extraction, plants were cultivated axenically as described in (Funck et al., 2010). Germination was scored as radicle protrusion and seeds that did not germinate were excluded from the assessment of further development. Approximately fifty seeds of each genotype were placed in a common Petri dish (9 cm diameter) and per condition, 4 plates with seeds from different parental plants were analyzed. To monitor root growth, 4-day-old seedlings were transferred to vertically placed Petri dishes (15 cm diameter) containing 50 ml of ½ x MS medium with the indicated supplements and 15 g/l of agar. Per condition, two plates with six seedlings of each genotype were analyzed. Seedlings were excluded from the analysis if their roots did not reach half of the length of the longest root from the same genotype and plate.

For salt stress experiments, plants were cultivated on soil (“Einheitserde”, type P, Gebr. Patzer, Sinntal-Altengronau, Germany) in a climate chamber with a light period of 9 h with day/night temperatures of 21°C/17°C and 50% relative humidity at a photon flux density of 120 ± 10 µmol m^−2^ s^−1^ from metal halide lamps (Clean Ace MT400DL/BH, Iwasaki, Tokyo, Japan). To induce salt stress, individual 9 cm pots with a single plant were twice soaked from the bottom with 25 ml of a 0.3 M NaCl solution at a two-day interval. Control plants received the same amount of water. To induce light stress, the plants were exposed for 1 h to 1,000 ± 100 µmol m^−2^ s^−1^ from metal halide lamps (Powerstar HQI-E bulb, 400 W/D; Osram, Germany) placed approximately 30 cm above the rosettes. Excess heat was removed by a fan directed mainly against the lamps.

### Northern Blot Analysis

RNA isolation, Northern blotting and detection with DIG-labeled probes (DIG-probe synthesis kit, Roche, Basel, Switzerland) was carried out as described in (Funck et al., 2008; Funck et al., 2010). A *P5CS2*-specific probe comprising primarily the 3′-UTR sequence was produced with the primers described in (Strizhov et al., 1997).

### Pathogen Defense Assays

*Pseudomonas syringae* pv. *tomato* strain DC3000 was cultivated in NGA medium (5 g L^−1^ peptone, 3 g L^−1^ yeast extract, 2% (V/V) glycerol, pH 7) with 50 µg ml^−1^ rifampicin. The medium for the strain carrying the avrRPM1 gene on a plasmid contained additionally 50 µg ml^−1^ kanamycin. Pathogen growth assays were carried out essentially as described in (Debener et al., 1991). Briefly, an exponentially growing *P. syringae* culture was harvested by centrifugation, resuspended to the desired density in 10 mM MgCl_2_ and used to infiltrate 5 leaves per plant trough the stomata of the lower epidermis with a 1 ml syringe. Leaf samples for the determination of bacterial titers were collected immediately after infiltration as well as 2 and 4 days post inoculation. After brief rinsing, three disks from independent leaves were macerated in 10 mM MgCl_2_ and a series of 5-fold dilutions was spotted in triplicates on NGA plates supplemented with rifampicin.

### Pigment and Proline Content Determination

For pigment extraction, fresh or freeze-dried leaves were ground under dim light in liquid nitrogen and pigments were extracted in 80% acetone (for simplicity, the entire fresh weight of the leaves was considered to be water). Quantification of pigments was carried out by determining the OD of suitable dilutions of the crude extracts at 470 nm, 647 nm and 663 nm according to (Lichtenthaler, 1987). Proline content of leaves was determined by a modification of the assay originally developed by (Bates et al., 1973) as described in (Funck et al., 2008).

### Leaf Sap Osmolality and Ion Concentrations

Crude leaf extracts were produced by macerating fresh leaves for 30 s at 30 Hz in a Tissue Lyser (Eppendorf, Hilden, Germany), heating the slurry to 96°C followed by centrifugation for 10 min at 4°C and 25,000 *g*. The extracts were diluted with double-distilled water to be within the linear range for determination of osmolality or ion concentrations. Osmolality was measured with a freeze point osmometer (Osmomat 30, Gonotech GmbH, Berlin, Germany) with distilled water and a NaCl solution with 0.3 Osm kg^−1^ as references. Concentrations of individual ions in the leaf sap were determined by inductively coupled plasma optical emission spectroscopy (ICP-OES) as described in (Gruber et al., 2013).

### Measurement of Photosynthetic Parameters

The ImagingWin software and an Imaging PAM (Walz, Effeltrich, Germany) equipped with standard measuring head were used to record chlorophyll fluorescence and absorptivity of excised leaves placed with the maximal recommended distance to the camera on moist filter paper. F_0_ was determined after 5 min dark adaptation at measuring light intensity and frequency settings of 1. After an initial saturating flash to determine F_m_, the leaves were exposed for 8 min to actinic blue light (86 µmol m^−2^ s^−1^) prior to the determination of F’ and F_m_’ before and during an additional saturating flash for the calculation of ΦPSII ((F_m_’-F’)/F_m_’). Absorptivity of the leaves was estimated according to the recommendations of the manufacturer from the difference between pictures with red and near infrared illumination and used to calculate rates of linear electron transport, assuming an equal distribution of absorbed photons between PS II and PS I. The NPQ parameter for inducible non-photochemical quenching is calculated by the ImagingWin software as (F_m_-F_m_’)/F_m_’. Fluorescence intensities and absorptivity were averaged over 2 circular areas per leaf with 2 mm diameter each.

### Statistical Analysis

Unless otherwise mentioned, statistical analyses were performed in R (R core team, 2020). The effects of proline and NaCl addition to the medium on the proportion of germinated seeds and established seedlings of the two *p5cs2* mutants at day 10 was assessed by binomial generalized linear mixed models as implemented in the *glmer* function from the *lme4* package (Bates et al., 2015). Models included genotype and either proline concentration (0, 2 and 10 mM) or NaCl concentration (0, 50, 100 and 200 mM) as fixed effects, and plate identity as a random effect. Because germination and establishment of wildtype and *p5cs1-4* seeds was at or very near to 100%, they could not be included into the model. Therefore, Fisher’s exact test was used to compare the proportions of germinated seeds or established seedlings under each condition to the wildtype using the RealStatistics Excel add-in (Zaiontz, 2020).

The effects of proline and NaCl in the medium on root growth of wildtype and mutant genotypes were assessed by a Gaussian linear mixed model as implemented in the *lmer* function from the *lme4* package in R (Bates et al., 2015). Models included genotype (wildtype, *p5cs1-4*, *p5cs2-1*, and *p5cs2-2*), proline concentration (0 and 2 mM), NaCl concentration (0 and 150 mM) and their interactions as fixed effects, and plate identity as a random effect. Photosynthetic parameters were appropriately transformed to obtain normal distribution of the model residuals and homogeneity of variance before the analysis with linear models (*lm* function in R). Post hoc pairwise comparisons between treatments or between genotypes were performed using the *glht* function of the *multcomp* package (Hothorn et al., 2008).

Other physiological parameters and bacterial titers were analyzed by one-way or two-way ANOVA with Tukey’s HSD test for multiple post-hoc pairwise comparisons using SigmaPlot (V13).

## Results

### *P5CS1*:GFP and *P5CS2*:GFP Fusion Proteins Are Localized in the Cytosol

**Figure 1:**
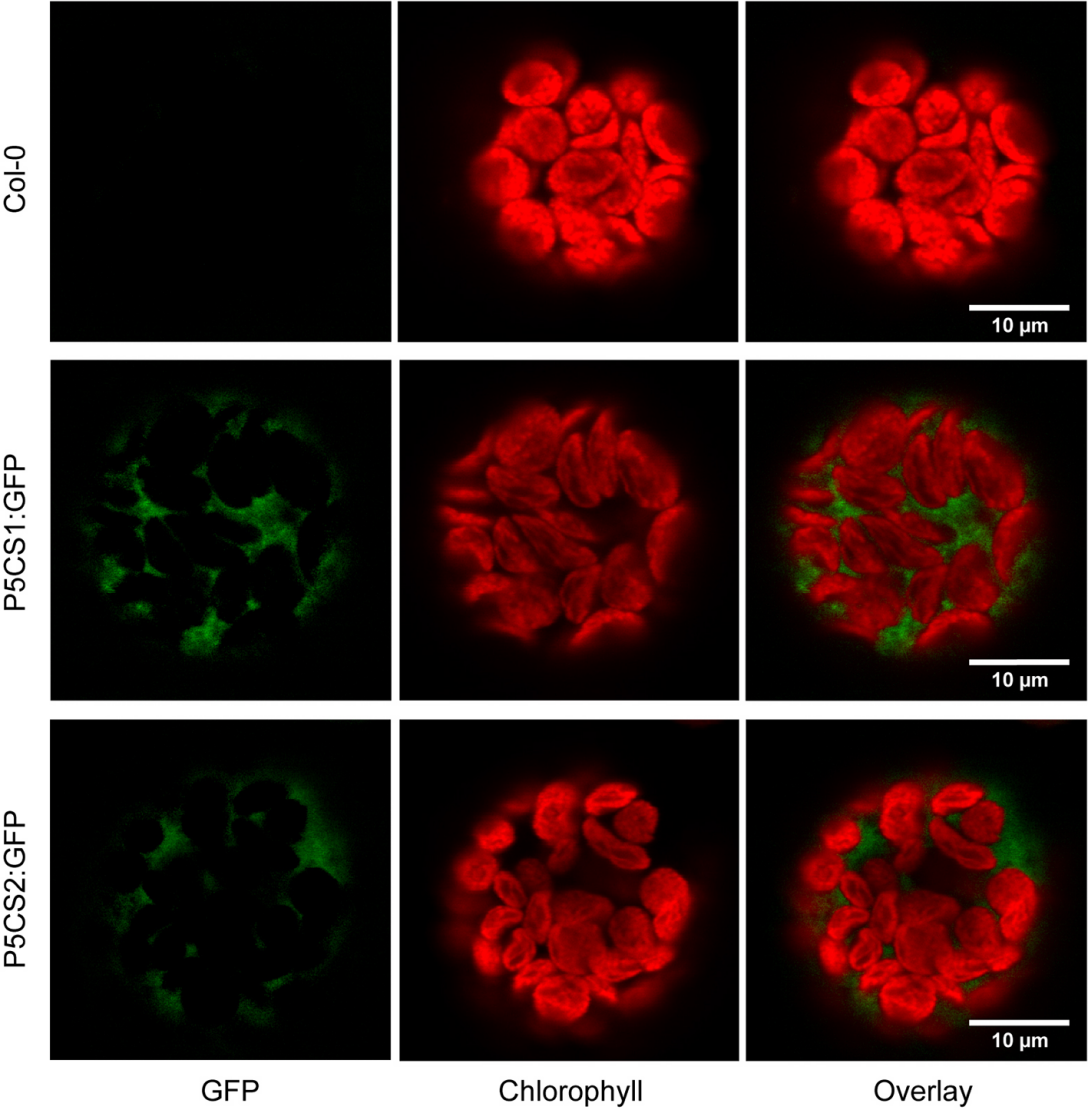
Subcellular localization of P5CS1 and P5CS2. GFP and chlorophyll fluorescence images of mesophyll protoplasts isolated from wildtype and P5CS1:GFP or P5CS2:GFP expressing plants. Spectrally resolved confocal fluorescence images with 20 channels spanning 490 to 668 nm emission wavelength were used for spectral unmixing with GFP and chlorophyll reference spectra. The GFP images and the overlay demonstrate that fluorescence emission from chloroplasts was entirely attributable to chlorophyll, and GFP fluorescence was exclusively detected in the cytosol. Compare with the simulated channel splitting mode images in **Supplementary Figure S1**.

A previous report suggested that both P5CS1 and P5CS2 from Arabidopsis can be localized in chloroplasts of stressed cells or isolated protoplasts despite the absence of recognizable transit peptides in the amino acid sequences (Székely et al., 2008). We re-visited the subcellular localization in transgenic plants stably expressing P5CS1:GFP and P5CS2:GFP fusion constructs under control of the constitutive Ubiquitin10 promoter. We used the Ubiquitin10 promoter, because it produces moderate expression levels and is less prone to silencing than other constitutive promoters (Grefen et al., 2010). When introduced into *p5cs1-4/p5cs1-4 p5cs2-1/P5CS2* sesquimutants, both constructs allowed the selection of plants in which all endogenous *P5CS* genes were inactivated by T-DNA insertions (**Supplementary Figure S1A**). With both constructs, we observed aggregation of the GFP fusion proteins in strongly expressing lines and frequently, the expression was partially or completely silenced (data not shown). Only lines with stable and moderate expression were used for microscopic analysis. In multi-wavelength confocal fluorescence images of leaf protoplasts, spectral unmixing showed that GFP signals from both P5CS1:GFP and P5CS2:GFP fusion proteins were exclusively detectable in the cytosol (**Figure 1** and **Supplementary Figure S1**). When the same images were processed to mimic channel splitting, it became evident that chlorophyll autofluorescence was also detectable in the GFP channel and generated the impression of dual localization of P5CS:GFP fusion proteins in the cytosol and in chloroplasts (**Supplementary Figures S1B, C**). Additionally, the images show that, despite identical microscope settings, chlorophyll fluorescence intensity in both the green and the red spectral range varied between individual protoplasts. The exclusive detection of both P5CS1:GFP and P5CS2:GFP in the cytosol indicates that sub-functionalization of the two P5CS isoforms in Arabidopsis is not depending on differential subcellular localization.

### Homozygous *p5cs2* Mutants Are Impaired in Germination and Seedling Development

**Figure 2:**
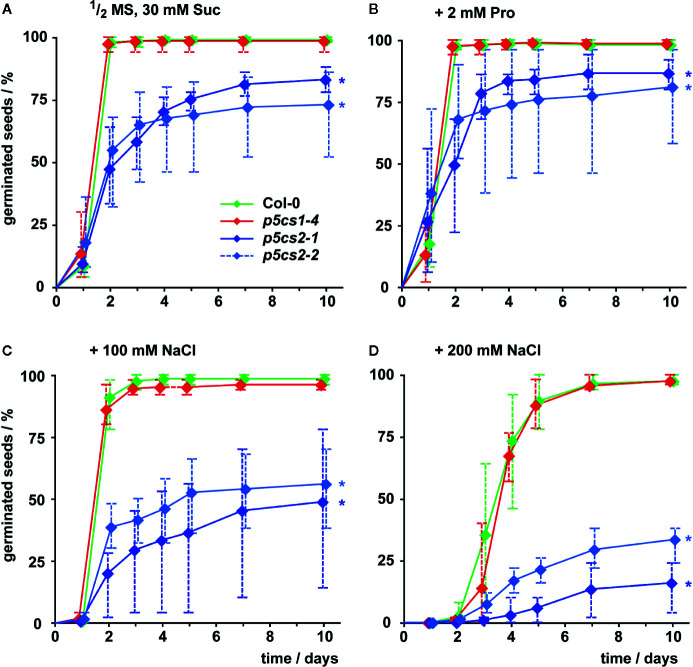
Influence of proline and NaCl on germination of *p5cs1* and *p5cs2* mutants. Germination (scored as radicle protrusion) was followed for 10 days after plating seeds on axenic half-strength MS medium containing 30 mM sucrose (Suc) without supplements **(A)**, supplemented with 2 mM proline **(B)**, 100 mM NaCl **(C)**, or 200 mM NaCl **(D)**. Diamonds represent mean proportions of germinated seeds from 4 batches, consisting of 50 seeds each, from individual parental plants. Whiskers extend to the highest and lowest values in each sample and asterisks indicate values that were significantly different from Col-0 at day 10 (p <0.05 by Fisher’s exact test).

To differentiate the roles of P5CS1 and P5CS2 in plant development and their contribution to stress tolerance, we compared single mutants lacking either isoform with wildtype plants under different cultivation conditions. Previous reports have shown that several /*p5cs1*/T-DNA insertion lines are phenotypically equivalent (Székely et al., 2008; Sharma et al., 2011) and therefore we chose to use the representative null-allele *p5cs1-4* for our experiments. Seed viability of two *p5cs2* null-mutants showed strong batch-to-batch variability and on average only 83% (*p5cs2-1*) and 73% (*p5cs2-2*) of the seeds had germinated after 10 d in sterile culture on half-strength Murashige and Skoog (1/2 MS) medium supplemented with 30 mM sucrose, compared to nearly 100% in *p5cs1-4* mutants and the wildtype (**Figure 2A**, **Supplementary Figure S2**). Nearly all wildtype and *p5cs1-4* seeds had germinated after two days, whereas a larger proportion among the seeds of both *p5cs2* mutant lines took three or more days to germinate. The presence of proline in the growth medium had no significant effect on the final proportion of germinated seeds of *p5cs2* mutants (**Figure 2B**, **Supplementary Table S1**). Similarly, application of mild salt stress (50 mM NaCl), which is sufficient to increase the expression of *P5CS1* in seedlings (see **Figure 5**), had no significant effect on germination (**Supplementary Figure S2B**). The presence of 100 mM and 200 mM NaCl in the medium delayed germination in all genotypes. While nearly all wildtype and *p5cs1-4* seeds eventually germinated, only 50% and 25% of the *p5cs2* mutant seeds had germinated after 10 days in the presence of 100 mM and 200 mM NaCl, respectively (**Figures 2C, D**).

**Figure 3:**
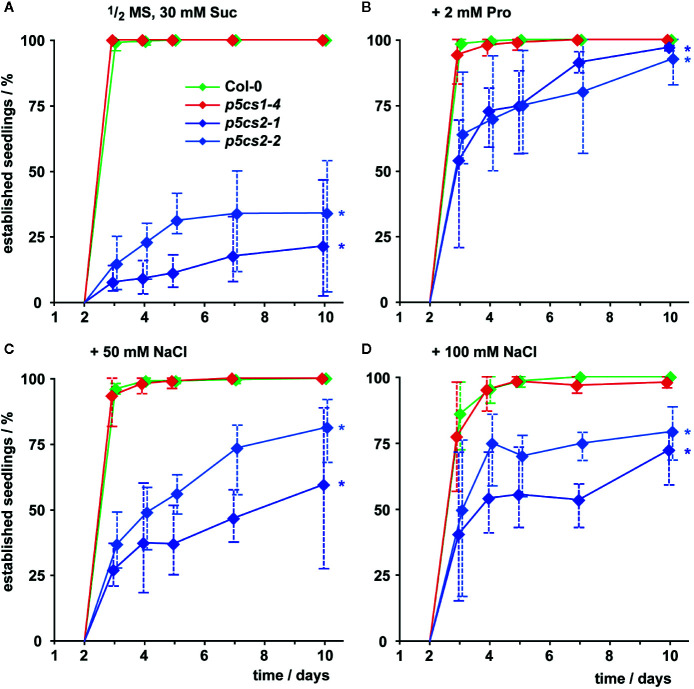
Seedling establishment of *p5cs1* and *p5cs2* mutants. Seedling establishment was determined between 2 and 10 days after plating of seeds on axenic half-strength MS medium containing 30 mM sucrose (Suc, **A**) supplemented with either 2 mM proline (Pro, **B**) or 50 mM **(C)** and 100 mM NaCl **(D)**. Seeds that had not germinated were excluded from the analysis and only seedlings with fully expanded, green cotyledons were scored as “established”. Diamonds represent mean proportions of established seedlings among the germinated seeds from 4 batches, consisting of 50 seeds each, from individual parental plants. Whiskers extend to the highest and lowest values in each sample and asterisks indicate values that were significantly different from Col-0 at day 10 (p <0.05 by Fisher’s exact test).

In the presence of external proline, nearly all successfully germinated seedlings of all four genotypes developed elongated hypocotyls and green, expanded cotyledons (which we scored as successful seedling establishment). These seedlings continued to grow and developed true leaves later on (**Figure 3**, **Supplementary Figure S2**). Compared to wildtype and *p5cs1* mutant seedlings, development of *p5cs2* mutant seedlings was a few days delayed during this early phase of development even in the presence of external proline. Without external supply of proline, only around 25% of the *p5cs2* mutant seedlings established successfully, despite the presence of 30 mM sucrose in the medium to promote germination and development (**Figure 3A**, **Supplementary Figure S2D**). The presence of 50 or 100 mM NaCl in the cultivation medium also significantly increased the proportion of *p5cs2* seedlings that successfully de-etiolated and expanded their cotyledons (**Supplementary Table S1**). At 200 mM NaCl, seedlings of all four genotypes failed to establish (**Supplementary Figure S2D**).

**Figure 4:**
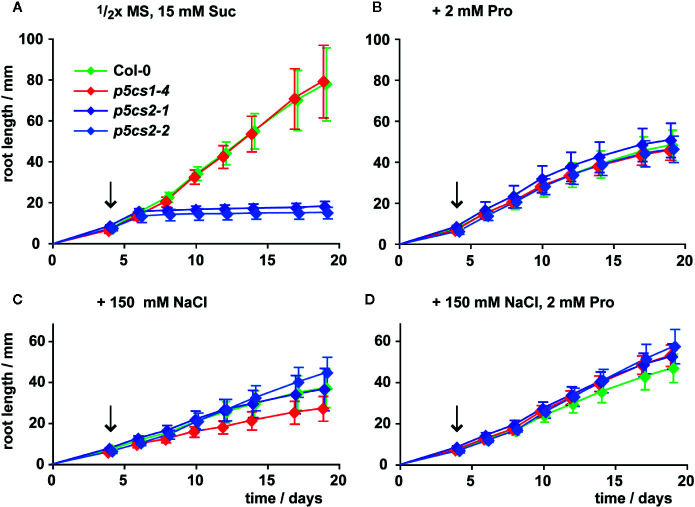
Influence of proline and NaCl on root growth of *p5cs1* and *p5cs2* mutants. Seedlings were pre-cultivated for 4 days on plates with half-strength MS medium supplemented with 15 mM sucrose (Suc) and 2 mM proline (Pro) to support establishment of *p5cs2* mutant seedlings. Then, established seedlings were transferred to vertical plates containing half-strength MS medium with 15 mM Suc without supplements **(A)**, or supplemented with 2 mM Pro **(B)**, 150 mM NaCl **(C)**, or both **(D)**. Arrows indicate time point of seedling transfer, after which root length was monitored for further 15 days. Diamonds represent the mean ± SD of 5 to 10 seedlings per genotype that developed continuously until day 19. The response of *p5cs2* mutants to the individual or combined treatments was significantly different from that of wildtype plants and *p5cs1-4* mutants (**Supplementary Table S2**).

Székely et al. (2008) described enhanced sensitivity of root elongation to NaCl stress in *p5cs1* mutants. Therefore, we also compared the effects of proline, NaCl, or both on root growth of *p5cs2* mutants, wildtype and *p5cs1-4* seedlings (**Figure 4**, **Supplementary Figure S3**, **Supplementary Table S2**). To minimize the influence of differences in establishment, all seedlings were initially germinated in the presence of 2 mM proline and transferred to fresh plates 4 days after sowing. When transferred to plates without external proline, roots of wildtype seedlings and *p5cs1-4* mutants elongated at a nearly linear rate to 8 cm on day 19 after plating (**Figure 4A**). Roots of *p5cs2* mutants continued to grow at the same rate as wildtype and *p5cs1-4* roots for 2 to 3 days, but then nearly completely stopped growing. When the seedlings were transferred to plates containing 2 mM proline, root growth of wildtype and *p5cs1-4* seedlings was reduced to approximately 60%. In contrast, the presence of 2 mM proline stimulated root growth of *p5cs2* mutants resulting in similar rates of root elongation in seedlings of all genotypes (**Figure 4B**). Similarly, supplementing the medium with 100 or 150 mM NaCl stimulated root growth of *p5cs2* mutants, but inhibited root growth of wildtype seedlings and even more of *p5cs1-4* mutants (**Figure 4C**, **Supplementary Figure S3**). The simultaneous supply with 2 mM proline slightly reduced the inhibitory effect of 150 mM NaCl in wildtype plants and /*p5cs1-4*/ mutants (**Figure 4D**).

In summary, both 2 mM proline or 150 mM NaCl had opposite effects on *p5cs2* mutants compared to wildtype plants or *p5cs1* mutants. On medium with 200 mM NaCl, which was lethal during germination (**Supplementary Figure S2D**), some of the transferred seedlings survived, but root growth was very poor in all genotypes (**Supplementary Figure S3**).

### The Influence of *p5cs* Mutations on Transcript Levels of Proline Metabolism-Related Genes Is Weak

**Figure 5:**
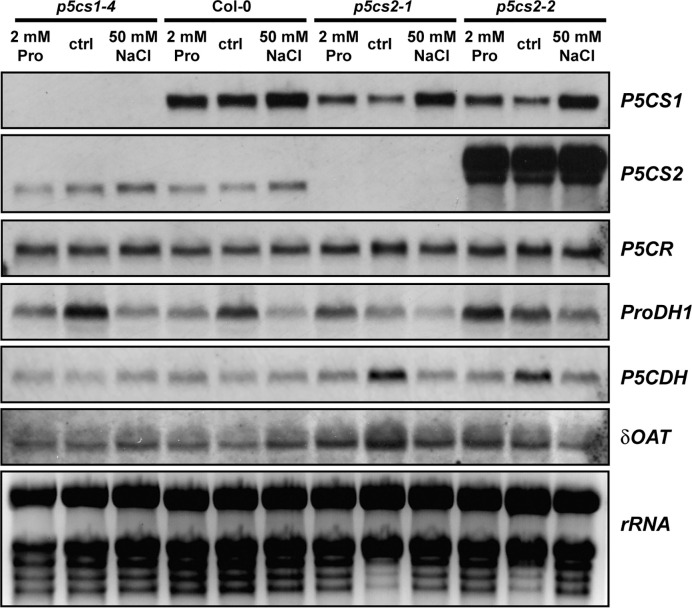
Transcript levels of proline metabolism-related genes. Wildtype (Col-0) seedlings and *p5cs1* or *p5cs2* mutants were harvested after 2 weeks of growth on half-strength MS medium with 30 mM sucrose (ctrl) supplemented with 50 mM NaCl or 2 mM proline (Pro). Total RNA was extracted and subjected to northern blotting by consecutively hybridizing the same membrane with specific probes for the genes indicated to the right of the panels. Ethidium bromide stained *rRNA* is shown as a loading control in the last panel. Two additional sets of Northern blots from independent experiments gave very similar results for all genes except *ProDH1* (See **Supplementary Figure S4**). For full names of the analyzed genes, please refer to the main text.

To determine if a mutation in one isoform of *P5CS* was compensated by altered expression of other enzymes involved in proline metabolism, we compared transcript levels by Northern blot analysis (**Figure 5**). The plants were cultivated for 2 weeks on half-strength MS medium supplemented with 30 mM sucrose and 50 mM NaCl or 2 mM proline. In *p5cs1-4* and *p5cs2-1* mutants, no transcripts of the corresponding genes were detected, while we detected large amounts of aberrant *P5CS2* transcripts in *p5cs2-2* mutants with a 3′UTR-specific probe. As expected, 50 mM NaCl induced *P5CS1* expression and repressed *ProDH1*-expression, but this effect was independent of *p5cs1* or *p5cs2* mutations. *P5CS1* transcript levels did not show differences between wildtype and *p5cs2* mutant seedlings, irrespective of the cultivation conditions. Transcript levels of *ProDH1* showed strong variability in all genotypes and under all conditions, but except for the repression by NaCl, no consistent pattern was detectable across the three repetitions of the experiment (**Supplementary Figure S4**). *P5C dehydrogenase (P5CDH)* reproducibly showed the highest transcript levels in *p5cs2* mutants in the absence of proline or NaCl. This may be a consequence of the developmental arrest during de-etiolation of *p5cs2* mutant seedlings. In none of the conditions and genotypes tested, a strong change in the level of *P5CR* or *Ornithine-δ-aminotransferase (δOAT)* transcripts was observed.

### *p5cs2* Mutants Are Not Impaired in Defense Against Virulent or Avirulent *Pseudomonas syringae* Strains

In Arabidopsis, *P5CS2* is considered as the “housekeeping” isoform with a prominent contribution to proline biosynthesis in rapidly growing tissues. Additionally, Fabro et al. (2004) reported a specific upregulation of *P5CS2* expression during the hypersensitive response in Arabidopsis leaves after infection with incompatible strains of *Pseudomonas syringae* pv. *tomato*. To test for a contribution of *P5CS1* and *P5CS2* to defense responses, we compared the pathogen susceptibility between wildtype plants and *p5cs1-4* or *p5cs2* mutants. We infected mature leaves of wildtype, *p5cs1-4* and *p5cs2* plants with virulent *P. syringae* DC3000 and with avirulent *P. syringae* DC3000 avrRpm1 (**Supplementary Figure S5**). The virulent bacteria multiplied more than 300-fold during the first two days, whereas the titers of viable bacteria started to decline between day two and day four, when disease symptoms became macroscopically visible. The avirulent, Rpm1-expressing bacteria multiplied less than 100-fold during the first two days and the plants started to show signs of HR-based defense already at that time (data not shown). Although the avirulent bacteria multiplied three to six times between day two and day four, they did not reach the same titers as the virulent bacteria. We did not observe significant differences in the bacterial titer between wildtype plants and *p5cs1-4* or *p5cs2* mutants infected with virulent *P. syringae* DC3000 (**Supplementary Table S3**). The avirulent *P. syringae* DC3000 avrRpm1 reached a slightly higher titer in *p5cs1-4* mutants and a slightly lower cell density in *p5cs2-1* mutant leaves, but in both mutants, the bacterial cell density differed less than a factor of 2 from the one reached in wildtype leaves. Obviously, the lack of *P5CS2* induction in late phases of an infection with avirulent bacteria did not compromise the defense in *p5cs2-1* mutants.

### *p5cs2* Mutants Are More Tolerant to NaCl Stress Than *p5cs1* Mutants or Wildtype Plants

**Figure 6:**
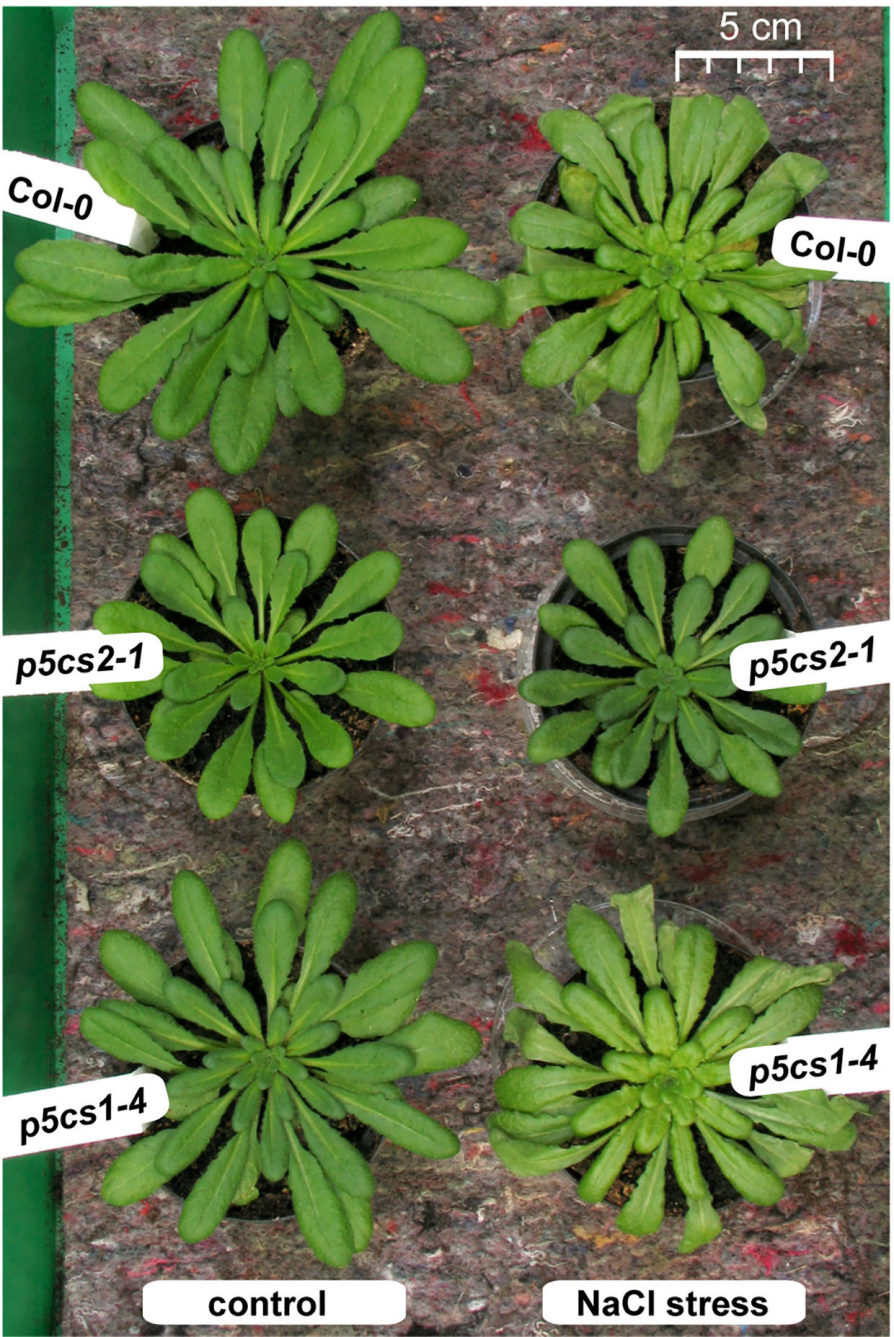
Phenotype of *p5cs* mutant plants after salt stress. Six-week-old plants were stressed 4 and 2 days before the picture was taken with 25 ml per pot of a 300 mM NaCl solution whereas control plants received normal tap water. Note that the left half of the picture with the control plants was already published as **Supplementary Figure S4** in (Funck et al., 2012) to illustrate the reduced rosette size of *p5cs2-1* mutants.

**Figure 7:**
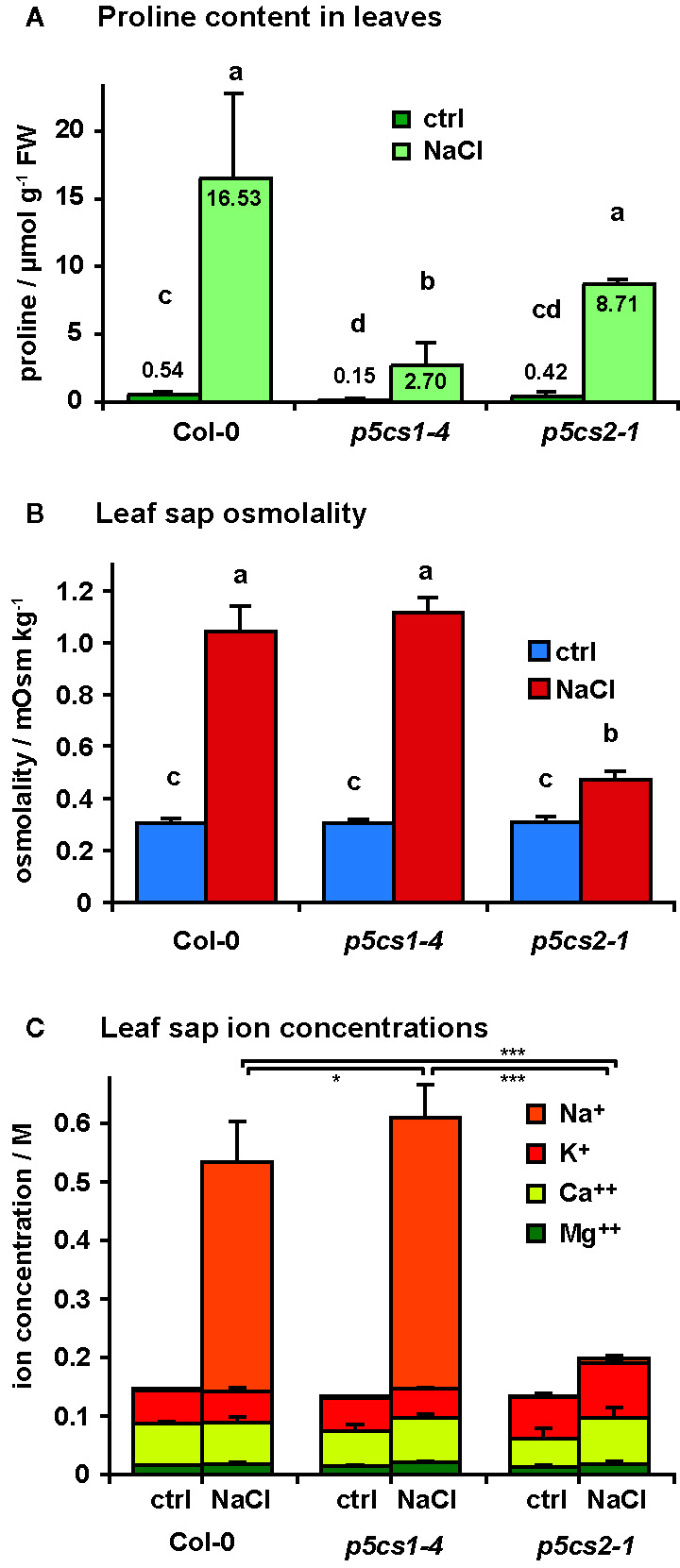
Salt accumulation and osmotic adjustment in *p5cs1* and *p5cs2* mutants. Six-week-old plants were stressed with 300 mM NaCl 4 and 2 days before the analysis of free proline content **(A)**, leaf sap osmolality **(B)**, and leaf sap cation concentrations **(C)**. Bars represent the average +SD of 4 or 5 replicates per condition and genotype. Two-way ANOVA detected significant differences depending on treatment and genotype in all three datasets (**Supplementary Table S4**). Leaf sap osmolality and Na^+^ concentrations under NaCl stress were indistinguishable between Col-0 and *p5cs1-4* mutants, but significantly lower in *p5cs2-1* mutants. Different letters above the columns in **(A)** and **(B)** indicate significantly different values (p < 0.05). Asterisks in **(C)** indicate significant difference between genotypes under NaCl stress (*, ***: p < 0.05 and < 0.001, respectively, see **Supplementary Table S4**).

Since we found no indication of altered susceptibility to bacterial infection in mature *p5cs1-4* and *p5cs2* mutants, we focused on their response to abiotic stress. To induce salt stress, we watered six-week-old plants twice with 300 mM NaCl solution, 4 and 2 days before sampling and analysis. This treatment caused the collapse of the oldest leaves of wildtype and *p5cs1-4* mutant plants and a slight curling and chlorosis of the younger leaves (**Figure 6**). Surprisingly, *p5cs2* mutants showed no visible symptoms of stress. Free proline content of the unstressed control plants was slightly lower in *p5cs2* mutants than in wildtype plants and significantly reduced in *p5cs1-4* mutants (**Figure 7A**, **Supplementary Table S4**). In response to stress, wildtype leaves showed an approximately 30-fold increase in proline content, reaching 16.5 µmol g^−1^ FW. While *p5cs2-1* mutants still reached 8.7 µmol g^−1^ FW, *p5cs1-4* were the least able to induce proline accumulation, reaching only 2.7 µmol g^−1^ FW. To get an idea of the importance of proline accumulation in osmotic adjustment, we determined the osmolality of crude, boiled leaf extracts. Under control conditions, wildtype leaf extract had an osmolality of 303 mOsm and *p5cs1-4* or *p5cs2-1* mutants were not significantly different (**Figure 7B**, **Supplementary Table S4**). After 4 days of NaCl stress, the osmolality increased to 1042 mOsm in wildtype and 1117 mOsm in *p5cs1-4* leaves, while *p5cs2-1* mutant leaves showed significantly lower values (472 mOsm), demonstrating that the extent of proline accumulation was not correlated to the overall osmotic adjustment.

Since changes in proline content could not account for the large differences in leaf sap osmolality, we analyzed the concentrations of different cations in the leaf sap (**Figure 7C**). In leaf sap of both wildtype and *p5cs1-4* mutant plants, an increase by more than 350 mM Na^+^ became evident after 4 days of salt treatment, whereas the Na^+^ concentration in *p5cs2-1* leaves did not increase significantly (1.3 mM in control conditions and 7.6 mM in NaCl stressed plants). The other analyzed ions (K^+^, Ca^++^ and Mg^++^) did not show major changes. The sum of all cation concentrations accounted for roughly 50% of the leaf sap osmolality in all three genotypes under both stress and non-stress conditions, indicating that the contribution of uncharged solutes to total osmolality was presumably rather small.

**Figure 8:**
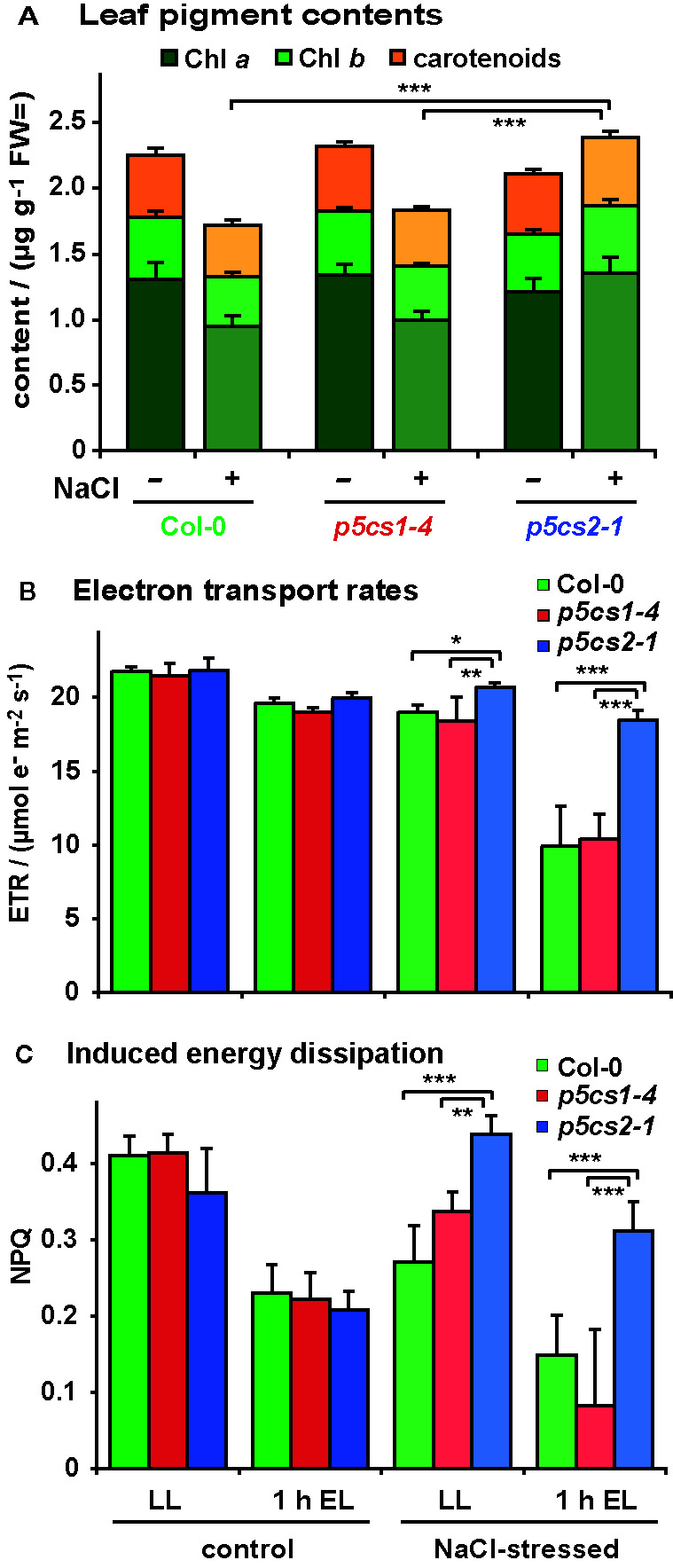
Influence of salt stress and excess light on photosynthetic performance of *p5cs1* and *p5cs2* mutants. Six-week-old plants were stressed with 300 mM NaCl 4 and 2 days before the analysis. **(A)** Pigment content of mature leaves. **(B)** Chlorophyll fluorescence analysis combined with red and near infrared reflection measurements was used to estimate electron transport rates prior to and after 1 h exposure to excess light stress (EL, 1,000 ± 100 µmol photons m^−2^ s^−1^). **(C)** Inducible energy dissipation (NPQ) measured on the same leaves as in **(B)**. Bars represent the average +SD of 4 or 5 replicates per condition and genotype. ANOVA analysis revealed significant differences depending on treatment, genotype and treatment x genotype interaction in all three data sets (**Supplementary Tables S5**, **S6**). Pairwise comparisons showed that NaCl stress caused reduced pigment contents in Col-0 and *p5cs1-4* mutants, but increased pigment contents in *p5cs2-1* mutants. Both NaCl stress and excess light treatment reduced ETR in all genotypes, but *p5cs2-1* mutants were significantly less affected. Inducible energy dissipation (NPQ) was decreased by NaCl stress and excess light treatment in Col-0 and *p5cs1-4* mutants, but increased by NaCl stress in *p5cs2-1* mutants. Asterisks indicate significant differences between genotypes within one condition (*, **, ***: p < 0.5, < 0.01, and < 0.001, respectively).

To determine if the brighter color of the stressed wildtype and *p5cs1-4* plants was the result of pigment breakdown, we determined the chlorophyll (Chl) and carotenoid content of the leaves. In the absence of stress, pigment content was indistinguishable between all three genotypes (**Figure 8A** and **Supplementary Table S5**). After stress, leaves of wildtype and *p5cs1-4* plants showed an approximately 20% decrease in total pigment content, whereby the decrease of Chl *a* was more pronounced than Chl *b* or total carotenoids. Consequently, the Chl *a*/Chl *b* ratio was significantly reduced by NaCl stress in wildtype and *p5cs1-4* plants. In accordance with the visual appearance, the total pigment content did not change much in *p5cs2-1* mutant leaves. The slight increase in pigment content per fresh weight in *p5cs2-1* mutants was due to a reduced water content, as evidenced in parallel experiments, in which pigment contents were determined on a dry weight basis from freeze-dried leaves (data not shown).

We also analyzed the effect of NaCl stress and altered pigmentation on the performance of the photosynthetic electron transport by *in vivo* Chl fluorescence imaging (**Figures 8B, C**; **Supplementary Table S6**). Székely et al. (2008) had observed increased production of reactive oxygen species in leaves of NaCl stressed *p5cs1* mutants. Chloroplasts are known as the main source for reactive oxygen species in illuminated plant cells, therefore we wondered if the accumulation of reactive oxygen species in *p5cs1* mutants could be caused by deteriorated photosynthetic electron transport. Since NaCl stress alone induced only minor changes in photosynthetic parameters, we additionally exposed the plants for 1 h to excess light stress (1,000 ± 100 µE) to enhance potential differences in stress tolerance. In the absence of NaCl stress, the photosynthetic electron transport rate (ETR) decreased by approximately 15% in all lines after light stress (**Figure 8B**). NaCl-stressed plants showed a similar reduction in ETR as light-stressed control plants, whereby *p5cs2-1* plants showed the smallest decrease in ETR. When NaCl-stressed plants were exposed to excess light stress, ETR dropped by 41% and 35% in wildtype and *p5cs1-4* leaves, respectively, whereas the decline in leaves of *p5cs2-1* mutants was only 13%. Absorptivity of photosynthetically active radiation (PAR), the maximum photochemical efficiency of PS II (F_v_/F_m_) and the photochemical efficiency in the light acclimated state (Φ_PSII_) showed nearly identical variations as ETR in dependence on genotype and treatment (**Supplementary Figure S6**). Similar results in experiments with *p5cs2-2* mutant plants confirmed the reduced sensitivity of *p5cs2* mutants to combined NaCl and excess light stress. In a minority of the repetitions of the NaCl stress experiment, an increased sensitivity of *p5cs1-4* mutants compared to wildtype plants was observed (data not shown). Overall, the visual appearance, ion and pigment content as well as the analysis of photosynthesis indicated that the tolerance towards NaCl stress was improved in *p5cs2* mutants, rendering them less susceptible to combined NaCl and excess light stress.

## Discussion

We used Arabidopsis *p5cs1* and *p5cs2* mutants to get a better understanding of the function of proline biosynthesis and accumulation in the stress tolerance of plants. The data presented here demonstrate that the two P5CS isoforms have different functions in stress defense, although both contribute to stress-induced proline accumulation and our results indicate that they are both localized in the cytosol. P5CS1 activity mediated the major part of proline accumulation, whereas P5CS2 activity was required for normal seedling development and especially root growth under non-stressed conditions. The developmental defects of *p5cs2* mutants were attenuated by external proline or mild salt stress and unexpectedly, the absence of P5CS2 made the plants more tolerant to salt stress and prevented the accumulation of Na^+^ ions in the leaves.

By careful spectral analysis of P5CS1:GFP and P5CS2:GFP expressing protoplasts (**Figure 1** and **Supplementary Figure S1B**), we did not find any evidence for chloroplast localization of P5CS1 or P5CS2, as it had been suggested in an earlier report (Székely et al., 2008). In our plants, the GFP fusion constructs consisted of a constitutive promoter and the cDNAs of *P5CS1* or *P5CS2*, whereas Székely et al. (2008) used the endogenous promoters and the full gene sequences. Complementation of *p5cs1-4/p5cs2-1* double mutants by our GFP constructs demonstrated that they confer functional P5CS1 or P5CS2 expression (**Supplementary Figure S1A**). We have previously demonstrated the utility of spectrally resolved fluorescence imaging to resolve ambiguous reports on dual localization of proteins in Arabidopsis (Huber et al., 2019). Especially when the GFP signals are weak and the experimental conditions are likely to influence chlorophyll fluorescence, as *e.g.* during the isolation of protoplasts, classical channel separation cannot efficiently distinguish between the small green component of chlorophyll autofluorescence and true GFP signals. Because Székely et al. (2008) used exclusively wavelength-based separation of GFP and chlorophyll fluorescence, and did not include pictures of wildtype cells, it remains ambiguous if the signals they detected in chloroplasts were true GFP signals. For Arabidopsis, our spectrally deconvoluted confocal images of protoplasts expressing P5CS1:GFP or P5CS2:GFP fusion proteins, together with the exclusively cytosolic localization of P5CR (Funck et al., 2012), strongly indicate that plastids do not contribute to P5CS-mediated proline biosynthesis. Contrasting reports on the subcellular localization of P5CS and P5CR in other plant species underline that this topic requires further attention (Rayapati et al., 1989; Szoke et al., 1992; Murahama et al., 2001; Kim and Nam, 2013).

Previous work had demonstrated that the T-DNA insertions in the two *p5cs2* mutant lines prevent the formation of functional *P5CS2* transcripts and cause conditional embryo lethality, especially when mutant seeds develop side by side with wildtype and heterozygous seeds in the same silique (Székely et al., 2008; Mattioli et al., 2009; Funck et al., 2012). *In vitro* cultivation of immature seeds allowed the rescue of fertile, homozygous *p5cs2* mutants (Funck et al., 2012). The seeds harvested from homozygous *p5cs2* mutants showed strong batch-to-batch variation in vitality, presumably depending on the physiological state of the parent plant (**Figure 2**). On average, seeds from *p5cs2* mutants germinated more slowly than wildtype seeds and the proportion of non-viable seeds was higher, irrespective of the presence of external proline in the medium. After germination, *p5cs2* mutant seedlings frequently failed to de-etiolate and establish vegetative growth in axenic culture in the absence of stress (**Figure 3**). Despite the presence of 15 mM sucrose in our cultivation medium, roots of *p5cs2* mutants barely elongated (**Figure 4**). Previous studies have highlighted the requirement of proline for the stimulation of cell division and growth in the root meristem (Wang et al., 2014; Biancucci et al., 2015). Supplementing the growth medium with 2 or 10 mM proline improved development of *p5cs2* seedlings, demonstrating the predominant role of P5CS2 in providing proline for growth and development. In contrast, external proline inhibited root growth of wildtype and *p5cs1* mutant seedlings. Inhibitory effects of external proline on plants have been described previously and underline the importance of proline homeostasis for optimal growth and development (Bonner et al., 1996; Hellmann et al., 2000; Hare et al., 2002).

An additional function of P5CS2 in defense against an incompatible pathovar of *P. syringae* had been proposed based on the upregulation of *P5CS2* expression in late phases of the hypersensitive defense response (Fabro et al., 2004). Other reports suggested that rather than proline biosynthesis, proline degradation by ProDH and the associated production of reactive oxygen species was important for pathogen defense (Miller et al., 2009; Monteoliva et al., 2014). In our experiments, disease symptoms and pathogen proliferation were very similar in wildtype plants and *p5cs2* or *p5cs1* mutants, irrespective of the use of compatible or incompatible *P. syringae* strains (**Supplementary Figure S5**). Therefore, it appears that *P5CS2* induction and proline accumulation are rather consequences of a HR-based defense than an active part of the defense mechanism.

In contrast to unchanged sensitivity of *p5cs1-4* and *p5cs2-1* mutants to biotic stress, their differential tolerance to abiotic stress, specifically NaCl-induced salt stress, revealed unexpected functions of P5CS2 in stress defense. Confirming previous studies, *p5cs1-4* mutants showed strongly reduced proline accumulation in response to NaCl stress, indicating that P5CS1 mediates the major part of proline accumulation in leaves (**Figure 7A**). P5CS1-mediated proline accumulation had been found to limit the production of reactive oxygen species in leaves and was proposed to provide an energy source (by means of proline transport and degradation) for sustained root growth during osmotic stress (Székely et al., 2008; Sharma et al., 2011). The presence of sucrose as a carbon and energy source in our culture medium may explain why the tolerance of *p5cs1-4* mutants to NaCl stress was only slightly compromised in our sterile culture setup. However, also soil-grown *p5cs1-4* mutants were phenotypically indistinguishable from wildtype plants under non-stressed conditions and despite the strongly reduced proline accumulation, they showed nearly unchanged stress sensitivity in our experiments.

In contrast, loss of *P5CS2* expression in the *p5cs2* mutants impaired their growth under non-stressed conditions, whereas mild salt stress improved seedling development, but not germination, of *p5cs2* mutants similar to the external supply of proline and abolished the differences to wildtype seedlings in root growth (**Figures 3**, **4**). The induction of *P5CS1* expression by NaCl and the resulting increase in proline content probably explain why mild salt stress and external proline both had a positive effect on the development of *p5cs2* seedlings (**Figures 5**, **7**). Northern blot analyses of *p5cs2* mutants grown from mature seeds revealed that *p5cs2-1* contained no detectable transcripts of the 3′-part of the *P5CS2* gene, while *p5cs2-2* mutants contained increased levels of aberrant, longer transcripts (**Figure 5**). These are presumably hybrid molecules with the 5′-end derived from the T-DNA inserted in the second exon of *P5CS2*. Both *p5cs2* mutant lines showed a similar increase of *P5CS1* transcript levels in response to NaCl treatment as wildtype seedlings. *Vice versa*, the expression of *P5CS2* was unaffected by the mutation in *p5cs1-4*, indicating little or no crosstalk or feedback in the regulation of expression of the two *P5CS* genes. The changes in transcript levels of other proline metabolism-related genes in response to NaCl or external proline also were very similar to wildtype plants in both *p5cs1* and *p5cs2* mutants.

Analyzing the rosettes of mature, soil grown plants, we found no consistent evidence for a contribution of *P5CS1* to the tolerance towards short-term salt stress, although in a subset of the experiments, *p5cs1-4* mutant plants were more severely affected by the stress treatment. Overall, NaCl accumulation, pigment loss and inhibition of photosynthetic electron transport were not significantly different between wildtype plants and *p5cs1-4* mutants, despite the strongly reduced proline accumulation in *p5cs1-4* mutants (**Figures 7**, **8**, **Supplementary Figure S6**). Notably, the reduced proline content did not result in an altered osmolality of crude leaf extracts from *p5cs1-4* mutants (**Figure 7B**), supporting previous estimations that the function of proline accumulation cannot be primarily the osmotic adjustment during stress (Hare and Cress, 1997; Forlani et al., 2019b). However, partitioning studies indicated that the predominant accumulation of proline in the cytosol and in chloroplasts of osmotically stressed potato leaves and in salt-stressed sugar beet may account for a relevant osmotic function of proline in these compartments (Büssis and Heineke, 1998; Hossain et al., 2017). Compartment-specific metabolite analyses in cold-stressed or high-light-exposed Arabidopsis rosettes confirmed that the distribution of proline within the cells changes in response to stress and is additionally dependent on changes in carbon metabolism (Fürtauer et al., 2016; Hörmiller et al., 2017; Küstner et al., 2019). Genetically encoded nanosensors for *in vivo* analysis of proline concentrations and osmotic potential, as they are already available for other amino acids and the subcellular redox potential, will need to be developed and used to determine the precise contribution of proline to osmotic adjustment (Bogner and Ludewig, 2007; Schwarzländer et al., 2016; Sanford and Palmer, 2017).

In sharp contrast to *p5cs1-4* mutants, soil-grown *p5cs2-1* mutant plants were much more tolerant to NaCl stress. Leaves of NaCl-treated *p5cs2-1* mutants showed no reduction in pigment content or photosynthetic electron transport (**Figure 8** and **Supplementary Figure S6**). This observation undermines the prevailing dogma that an increased content of compatible solutes is a direct measure and cause of stress tolerance, because proline accumulation was intermediate in *p5cs2-1* mutants compared to wildtype plants and *p5cs1-4* mutants. Stress perception was obviously not affected, since *p5cs2* mutants showed a similar degree of *P5CS1* induction as wildtype plants (**Figure 5**). The relative increase in proline content in *p5cs2-1* mutants (21-fold) was also intermediate between *p5cs1-4* mutants (19-fold) and wildtype plants (31-fold). Thus, other factors than overall proline synthesis or content must be the reason for the reduced stress sensitivity of *p5cs2-1* mutants.

The most striking difference between wildtype plants and *p5cs1-4* mutants on the one side and *p5cs2-1* mutants on the other side was observed in the osmolality and ion concentrations of crude leaf extracts after treatment with NaCl (**Figures 7B, C**). Wildtype plants and *p5cs1-4* mutants accumulated 390 and 460 mM Na^+^, respectively, in the leaves, whereas in leaves of *p5cs2-1* mutants the Na^+^ concentrations was not elevated. The largely unchanged levels of the other analyzed cations (K^+^, Ca^++^, Mg^++^) indicated that the stress symptoms observed in the leaves were mainly caused by osmotic effects or by toxicity of high Na^+^, and presumably also Cl^−^, concentrations. The presence of large amounts of Na^+^ ions in the rosettes of wildtype plants and *p5cs1-4* mutants after application of salt solution to the soil demonstrated that Na^+^ entered the roots and was efficiently transferred to the shoot. The mutation of *P5CS2* prevented the accumulation of Na^+^ in the leaves or delayed it beyond the time of our analyses and thereby protected the *p5cs2-1* mutants from Na^+^ toxicity.

As in many previous reports, especially on the genetic engineering of stress tolerance, the increased resistance of *p5cs2-1* mutant plants comes at the cost of reduced growth under non-stress conditions (Lawlor, 2013). We propose that three factors can, individually or in combination, explain the reduced salt accumulation and thus increased tolerance of *p5cs2* mutants and these hypotheses can be tested in future experiments: First, the smaller rosettes of *p5cs2* mutants (Funck et al., 2012) could result in lower transpiration rates and consequently a reduced uptake of water and solutes from the soil. Second, the shorter roots of *p5cs2* mutants under non-stressed conditions provide a lower contact area to the substrate with a reduced absolute number of membrane proteins, through which Na^+^ ions can enter the root cells. Third, the reduced proline synthesis and content especially in the meristematic tissue of the root tips, where *P5CS2* is predominantly expressed in wildtype plants (Székely et al., 2008), could pre-adapt the *p5cs2* mutants to prevent excessive Na^+^ uptake or transfer to the leaves. This could be achieved by increased expression of Na^+^/H^+^ antiporters like SOS1 and NHX1 or by limiting the entry of Na^+^ into the xylem by increased HKT1 expression (Zhang and Shi, 2013). Our preliminary analyses gave inconsistent results for the regulation of these genes in roots of *p5cs2-1* mutants. Future experiments will additionally show whether temporally or spatially confined suppression of *P5CS2* expression can be used to enhance salt tolerance without compromising plant productivity and fitness under non-stressful conditions.

## Data Availability Statement

The raw data supporting the conclusions of this article will be made available by the authors, without undue reservation.

## Author Contributions

DF designed the study, performed or supervised the majority of the experiments, and wrote the initial version of the manuscript. LB and LS performed the analyses of stressed plants. NvW performed the ion quantifications. MS contributed to the experimental design and the statistical analyses. All authors contributed to the article and approved the submitted version.

## Funding

This work was funded by the Excellence Initiative of the University of Konstanz (Young Scholar Fund). LS received a fellowship from the Heinz Böckler Foundation (390471).

## Conflict of Interest

The authors declare that the research was conducted in the absence of any commercial or financial relationships that could be construed as a potential conflict of interest.
