# Supplementary material for: Differential Contribution of P5CS Isoforms to Stress Tolerance in Arabidopsis
Source: Front Plant Sci. 2020 Sep 25;11:565134. doi: 10.3389/fpls.2020.565134 (PMC7545825; doi:10.3389/fpls.2020.565134)
Supplement: Supplementary file 1 [file DataSheet_1.pdf]

**Supplementary Table S1: Statistical analyses of germination and establishment**
Binomial generalized linear mixed models were used to compare different genotypes among each other as well as their response to the different media (Figures 2, 3 and Supplementary Figure S2). Germination and establishment of wildtype and *p5cs1-4* seedlings were too close to 100% to be included in the statistical analyses.

| **Influence of 0, 2, or 10 mM proline on germination of *p5cs2* mutants (Figures 2, S2)** | | | | | **Influence of 0, 50, 100, or 200 mM NaCl on germination of *p5cs2* mutants (Figures 2, S2)** | | | | |
|---|---|---|---|---|---|---|---|---|---|
| **Source of Variation** | **DF[a]** | **AIC[b]** | **LRT[c]** | **p-value** | **Source of Variation** | **DF[a]** | **AIC[b]** | **LRT[c]** | **p-value** |
| Supplement | 2 | 157.1 | 1.64 | 0.44 | Supplement | 3 | 298.5 | 23.89 | **<0.001** |
| Genotype (*p5cs2-1* vs *p5cs2-2*) | 1 | 170.3 | 12.84 | **<0.001** | Genotype (*p5cs2-1* vs *p5cs2-2*) | 1 | 284.6 | 5.96 | **0.02** |
| Supplement x genotype | 2 | 159.5 | 0.35 | 0.84 | Supplement x genotype | 3 | 280.6 | 21.93 | **<0.001** |

**Pairwise comparisons with General Linear Hypotheses**

| **Fixed factor** | **comparison** | **z-value** | **p-value** | **Fixed factor** | **comparison** | **z-value** | **p-value** |
|---|---|---|---|---|---|---|---|
| 0 mM proline | *p5cs2-1* vs *p5cs2-2* | -2.47 | 0.04 | *p5cs2-1* | 0 vs 50 mM NaCl | -1.59 | 0.40 |
| 2 mM proline | | -1.52 | 0.34 | *p5cs2-2* | | 0.78 | 0.92 |
| 10 mM proline | | -2.14 | 0.09 | *p5cs2-1* | 0 vs 100 mM NaCl | -4.09 | **<0.001** |
| | | | | *p5cs2-2* | | -1.92 | 0.22 |
| | | | | *p5cs2-1* | 0 vs 200 mM NaCl | -7.69 | **<0.001** |
| | | | | *p5cs2-2* | | -4.29 | **<0.001** |

| **Influence of 0, 2, or 10 mM proline on establishment of *p5cs2* mutants (Figures 3, S2)** | | | | | **Influence of 0, 50 or 100 mM NaCl on establishment of *p5cs2* mutants (Figure 3)** | | | | |
|---|---|---|---|---|---|---|---|---|---|
| **Source of Variation** | **DF[a]** | **AIC[b]** | **LRT[c]** | **p-value** | **Source of Variation** | **DF[a]** | **AIC[b]** | **LRT[c]** | **p-value** |
| Supplement | 2 | 199.3 | 22.01 | **<0.001** | Supplement | 2 | 199.3 | 12.21 | **0.002** |
| Genotype (*p5cs2-1* vs *p5cs2-2*) | 1 | 183.0 | 3.71 | 0.054 | Genotype (*p5cs2-1* vs *p5cs2-2*) | 1 | 223.2 | 34.11 | **<0.001** |
| Supplement x genotype | 2 | 181.3 | 13.82 | **<0.001** | Supplement x genotype | 2 | 191.1 | 0.26 | 0.88 |

**Pairwise comparisons with General Linear Hypotheses**

| **Fixed factor** | **comparison** | **z-value** | **p-value** | **Fixed factor** | **comparison** | **z-value** | **p-value** |
|---|---|---|---|---|---|---|---|
| 0 mM proline | *p5cs2-1* vs *p5cs2-2* | 3.77 | **<0.01** | 0 mM NaCl | *p5cs2-1* vs *p5cs2-2* | 3.77 | **<0.01** |
| 2 mM proline | | -1.58 | 0.49 | 50 mM NaCl | | 3.61 | **<0.01** |
| 10 mM proline | | -0.70 | 0.97 | 100 mM NaCl | | 2.48 | 0.08 |
| *p5cs2-1* | 0 vs 2 mM proline | 7.19 | **<0.001** | *p5cs2-1* | 0 vs 50 mM NaCl | 3.72 | **<0.01** |
| *p5cs2-2* | | 5.03 | **<0.001** | *p5cs2-2* | | 3.58 | **<0.01** |
| *p5cs2-1* | 0 vs 10 mM proline | 6.71 | **<0.001** | *p5cs2-1* | 0 vs 100 mM NaCl | 3.82 | **<0.001** |
| *p5cs2-2* | | 4.64 | **<0.001** | *p5cs2-2* | | 3.39 | **<0.01** |

[a] Degrees of freedom
[b] Akaike information criterion
[c] Likelihood ratio test

**Supplementary Table S2: Statistical analyses of root length**

The effects of proline and NaCl on root growth of wildtype and mutant genotypes were assessed by a Gaussian linear mixed model (Figure 4 and Supplementary Figure S3).

| **Influence of 0 or 2 mM proline and 0 or 150 mM NaCl on root length (Figures 4, S3)** | | | | |
|---|---|---|---|---|
| **Source of Variation** | **DF[a]** | **AIC[b]** | **LRT[c]** | **p-value** |
| Proline[d] | 1 | 2007.5 | 2.12 | 0.15 |
| NaCl[d] | 1 | 2006.0 | 0.60 | 0.445 |
| Genotype | 3 | 2028.0 | 26.61 | **<0.001** |
| Genotype x proline | 3 | 1930.3 | 46.46 | **<0.001** |
| Genotype x NaCl | 3 | 1973.6 | 89.83 | **<0.001** |
| Proline x NaCl | 1 | 1889.9 | 2.15 | 0.14 |
| Genotype x proline x NaCl | 3 | 1889.8 | 99.58 | **<0.001** |

| **Pairwise comparisons within fixed factors with General Linear Hypotheses** | | | | | | | |
|---|---|---|---|---|---|---|---|
| **Fixed factor** | **comparison** | **z-value** | **p-value** | **Fixed factor** | **Treatment** compared to 0 Pro, 0 NaCl | **z-value** | **p-value** |
| 0 Pro, 0 NaCl | Col-0 vs *p5cs1-4* | -0.42 | 0.96 | Col-0 | 2 Pro, 0 NaCl | 2.84 | **0.01** |
| | Col-0 vs *p5cs2-1* | 17.17 | **<0.001** | | 0 Pro, 150 NaCl | 4.75 | **<0.001** |
| | Col-0 vs *p5cs2-2* | 18.94 | **<0.001** | | 2 Pro, 150 NaCl | 3.73 | **<0.001** |
| 2 Pro, 0 NaCl | Col-0 vs *p5cs1-4* | 0.55 | 0.91 | *p5cs1-4* | 2 Pro, 0 NaCl | 3.22 | **<0.01** |
| | Col-0 vs *p5cs2-1* | -0.50 | 0.93 | | 0 Pro, 150 NaCl | 5.94 | **<0.001** |
| | Col-0 vs *p5cs2-2* | 0.42 | 0.95 | | 2 Pro, 150 NaCl | 3.21 | **<0.01** |
| 0 Pro, 150 NaCl | Col-0 vs *p5cs1-4* | 2.44 | **0.04** | *p5cs2-1* | 2 Pro, 0 NaCl | -3.09 | **<0.01** |
| | Col-0 vs *p5cs2-1* | 0.14 | 1.00 | | 0 Pro, 150 NaCl | -2.12 | 0.09 |
| | Col-0 vs *p5cs2-2* | -2.05 | 0.11 | | 2 Pro, 150 NaCl | -3.99 | **<0.001** |
| 2 Pro, 150 NaCl | Col-0 vs *p5cs1-4* | -1.32 | 0.42 | *p5cs2-2* | 2 Pro, 0 NaCl | -3.01 | **<0.01** |
| | Col-0 vs *p5cs2-1* | -1.81 | 0.18 | | 0 Pro, 150 NaCl | -3.46 | **<0.01** |
| | Col-0 vs *p5cs2-2* | -3.09 | **<0.01** | | 2 Pro, 150 NaCl | -4.92 | **<0.001** |

[a] Degrees of freedom

[b] Akaike information criterion

[c] Likelihood ratio test

[d] Overall effects of proline and NaCl were not significant, because the different genotypes responded in opposite directions

**Supplementary Table S3: Statistical analyses of bacterial titers after infection with virulent or avirulent *Pseudomonas syringae* pv tomato DC3000 bacteria**

**(A)** One-way ANOVA and of bacterial titers 0, 2 and 4 days after infection with virulent bacteria (Supplementary Figure S5A)

| **Source of variation** | **Degrees of freedom** | **Sum of squares** | | | **Mean squares** | | | **F-value** | | | **p-value** | | |
|---|---|---|---|---|---|---|---|---|---|---|---|---|---|
| Time | | Day 0 | Day 2 | Day 4 | Day 0 | Day 2 | Day 4 | Day 0 | Day 2 | Day 4 | Day 0 | Day 2 | Day 4 |
| Genotype | 3 | 2.29E+08 | 3.61E+14 | 2.74E+13 | 7.63E+07 | 1.20E+14 | 9.13E+12 | 3.15 | 1.01 | 2.05 | 0.07 | 0.42 | 0.16 |
| Error | 12 | 2.91E+08 | 1.42E+15 | 5.33E+13 | 2.43E+07 | 1.19E+14 | 4.44E+12 | | | | | | |
| Total | 15 | 5.20E+08 | 1.79E+15 | 8.07E+13 | | | | | | | | | |

**(B)** One-way ANOVA of bacterial titers 4 days after infection with avirulent, RPM1-expressing, bacteria (Supplementary Figure S5B)

| **Source of variation** | **Degrees of freedom** | **Sum of squares** | | | **Mean squares** | | | **F-value** | | | **p-value** | | |
|---|---|---|---|---|---|---|---|---|---|---|---|---|---|
| Time | | Day 0 | Day 2 | Day 4 | Day 0 | Day 2 | Day 4 | Day 0 | Day 2 | Day 4 | Day 0 | Day 2 | Day 4 |
| Genotype | 2 | 7.38E+06 | 4.69E+11 | 1.16E+13 | 3.69E+06 | 2.34E+11 | 5.79E+12 | 1.05 | 3.40 | 9.89 | 0.39 | 0.08 | **<0.01** |
| Error | 9 | 3.17E+07 | 6.21E+11 | 5.27E+12 | 3.53E+06 | 6.90E+10 | 5.86E+11 | | | | | | |
| Total | 11 | 3.91E+07 | 1.09E+12 | 1.69E+13 | | | | | | | | | |

**(C)** Pairwise comparisons of bacterial titers 4 days after infection with avirulent, RPM1-expressing, bacteria with Tukey's HSD test

| **Genotype pair** | **q-value** | **p-value** |
|---|---|---|
| Col-0 *vs. p5cs1-4* | 4.23 | **0.04** |
| Col-0 *vs. p5cs2-1* | 1.93 | 0.40 |
| *p5cs1-4 vs. p5cs2-1* | 6.16 | **<0.01** |

**Supplementary Table S4A: Statistical analyses of solutes in mature rosette leaves of wildtype plants and *p5cs1* or *p5cs2* mutants under non-stressed and stressed conditions.**

| Source of Variation | Degrees of freedom | Sum of squares | Mean squares | F-value | p-value |
|---|---|---|---|---|---|
| **2-way ANOVA of proline content[a] (Figure 7A)** | | | | | |
| Treatment | 1 | 11.26 | 11.26 | 423.2 | **<0.001** |
| Genotype | 2 | 1.54 | 0.77 | 29.0 | **<0.001** |
| Genotype x treatment | 2 | 0.02 | 0.01 | 0.4 | 0.69 |
| Residual | 17 | 0.45 | 0.03 | | |
| Total | 22 | 13.97 | 0.64 | | |
| **2-way ANOVA of leaf sap osmolality[b] (Figure 7B)** | | | | | |
| Treatment | 1 | 2.36 | 2.36 | 919.5 | **<0.001** |
| Genotype | 2 | 0.57 | 0.28 | 110.5 | **<0.001** |
| Genotype x treatment | 2 | 0.58 | 0.29 | 113.4 | **<0.001** |
| Residual | 23 | 0.06 | 0.003 | | |
| Total | 28 | 3.74 | 0.13 | | |

| **All pairwise comparisons with Tukey's HSD test** | | | | |
|---|---|---|---|---|
| | **Proline content[a]** | | **Osmolality** | |
| **comparison** | **q-value** | **p-value** | **q-value** | **p-value** |
| Col-0, ctrl vs *p5cs1-4*, ctrl | 1.69 | **<0.01** | 0.006 | 1.00 |
| Col-0, ctrl vs *p5cs2-1*, ctrl | 5.83 | 0.82 | 0.475 | 1.00 |
| Col-0, ctrl vs Col-0, NaCl | 15.29 | **<0.01** | 41.356 | **<0.001** |
| Col-0, ctrl vs *p5cs1-4*, NaCl | 11.72 | **<0.01** | 43.502 | **<0.001** |
| Col-0, ctrl vs *p5cs1-4*, NaCl | 6.61 | **<0.01** | 15.127 | **<0.001** |
| *p5cs1-4*, ctrl vs *p5cs2-1*, ctrl | 4.14 | 0.08 | 0.468 | 1.00 |
| *p5cs1-4*, ctrl vs Col-0, NaCl | 16.98 | **<0.01** | 41.350 | **<0.001** |
| *p5cs1-4*, ctrl vs *p5cs1-4*, NaCl | 13.28 | **<0.01** | 43.496 | **<0.001** |
| *p5cs1-4*, ctrl vs *p5cs2-1*, NaCl | 8.30 | **<0.01** | 15.121 | **<0.001** |
| *p5cs2-1*, ctrl vs Col-0, NaCl | 21.12 | **<0.01** | 40.881 | **<0.001** |
| *p5cs2-1*, ctrl vs *p5cs1-4*, NaCl | 17.11 | **<0.01** | 43.028 | **<0.001** |
| *p5cs2-1*, ctrl vs *p5cs2-1*, NaCl | 12.44 | **<0.01** | 14.679 | **<0.001** |
| Col-0, NaCl vs *p5cs1-4*, NaCl | 2.44 | **<0.01** | 2.146 | 0.66 |
| Col-0, NaCl vs *p5cs2-1*, NaCl | 8.68 | 0.53 | 23.864 | **<0.001** |
| *p5cs1-4*, NaCl vs *p5cs2-1*, NaCl | 5.59 | **0.01** | 25.888 | **<0.001** |

[a] values were log-transformed to obtain normal distribution and equal variance

[a] values were ^-0.2-transformed to obtain normal distribution and equal variance

**Supplementary Table S4B: Statistical analyses of solutes in mature rosette leaves of wildtype plants and *p5cs1* or *p5cs2* mutants under non-stressed and stressed conditions.**

| **2-way ANOVA of total cation concentration (Figure 7C)** | | | | | |
|---|---|---|---|---|---|
| **Source of Variation** | **Degrees of freedom** | **Sum of squares** | **Mean squares** | **F-value** | **p-value** |
| Treatment | 1 | 720175 | 720175 | 391.5 | **<0.001** |
| Genotype | 2 | 246765 | 123383 | 67.1 | **<0.001** |
| Genotype x treatment | 2 | 235513 | 117757 | 64.0 | **<0.001** |
| Residuals | 24 | 44150 | 1840 | | |
| Total | 29 | 1246603 | 42986 | | |

| **Pairwise comparisons between genotypes or treatments with Tukey's HSD test** | | | | |
|---|---|---|---|---|
| **Fixed Factor** | **Comparison** | **Diff. of Means** | **q-value** | **p-value** |
| Col-0 | ctrl vs NaCl | 388.2 | 20.24 | **<0.001** |
| *p5cs1*-4 | | 476.9 | 24.86 | **<0.001** |
| *p5cs2*-1 | | 64.6 | 3.37 | **0.03** |
| ctrl | Col-0 vs *p5cs1-4* | 12.53 | 0.653 | 0.89 |
| | Col-0 vs *p5cs2-1* | 12.69 | 0.661 | 0.89 |
| | *p5cs1-4* vs *p5cs2-1* | 0.15 | 0.008 | 1.00 |
| NaCl | Col-0 vs *p5cs1-4* | 76.16 | 3.970 | **0.03** |
| | Col-0 vs *p5cs2-1* | 336.32 | 17.534 | **<0.001** |
| | *p5cs1-4* vs *p5cs2-1* | 412.48 | 21.504 | **<0.001** |

**Supplementary Table S5: Statistical analyses of pigment contents and pigment ratios in mature rosette leaves of wildtype plants and *p5cs1* or *p5cs2* mutants under non-stressed and stressed conditions.**

| Source of Variation | Degrees of freedom | Sum of squares | Mean squares | F-value | p-value |
|---|---|---|---|---|---|
| **2-way ANOVA of total pigment contents (Figure 8A)** | | | | | |
| Treatment | 1 | 0.44 | 0.44 | 14.49 | **<0.001** |
| Genotype | 2 | 0.36 | 0.18 | 5.86 | **<0.01** |
| Treatment x genotype | 2 | 1.04 | 0.52 | 17.11 | **<0.001** |
| Residual | 24 | 0.73 | 0.03 | | |
| **2-way ANOVA of Chl *a* / Chl *b* ratio** | | | | | |
| Treatment | 1 | 0.44 | 0.44 | 14.49 | **<0.001** |
| Genotype | 2 | 0.36 | 0.18 | 5.86 | **<0.01** |
| Treatment x genotype | 2 | 1.04 | 0.52 | 17.11 | **<0.001** |
| Residual | 24 | 0.73 | 0.03 | | |

| **Pairwise comparisons [a]** | | **Total pigment content** | | **Chl *a* / Chl *b* ratio** | |
|---|---|---|---|---|---|
| **Fixed Factor** | **Comparison** | **q-value** | **p-value** | **q-value** | **p-value** |
| Col-0 | ctrl vs. NaCl | 6.79 | **<0.001** | 8.28 | **<0.001** |
| *p5cs1*-4 | | 6.17 | **<0.001** | 10.37 | **<0.001** |
| *p5cs2*-1 | | 3.64 | **0.02** | 2.93 | 0.05 |
| ctrl | Col-0 vs *p5cs1-4* | 0.85 | 0.82 | 0.48 | 0.94 |
| | Col-0 vs *p5cs2-1* | 1.84 | 0.41 | 0.32 | 0.97 |
| | *p5cs1-4* vs *p5cs2-1* | 2.69 | 0.16 | 0.17 | 0.99 |
| NaCl | Col-0 vs *p5cs1-4* | 1.46 | 0.56 | 2.57 | 0.19 |
| | Col-0 vs *p5cs2-1* | 8.58 | **<0.001** | 5.04 | **<0.01** |
| | *p5cs1-4* vs *p5cs2-1* | 7.12 | **<0.001** | 7.61 | **<0.001** |

| Source of Variation | Degrees of freedom | Sum of squares | Mean squares | F-value | p-value |
|---|---|---|---|---|---|
| **2-way ANOVA of Chl *a* content (Figure 8A)** | | | | | |
| Treatment | 1 | 0.256 | 0.256 | 25.66 | **<0.001** |
| Genotype | 2 | 0.136 | 0.068 | 6.83 | **<0.01** |
| Treatment x genotype | 2 | 0.412 | 0.206 | 20.62 | **<0.001** |
| Residual | 24 | 0.239 | 0.010 | | |
| **2-way ANOVA of Chl *b* content (Figure 8A)** | | | | | |
| Treatment | 1 | 0.008 | 0.008 | 5.23 | **0.03** |
| Genotype | 2 | 0.012 | 0.006 | 4.18 | **0.03** |
| Treatment x genotype | 2 | 0.040 | 0.020 | 13.85 | **<0.001** |
| Residual | 24 | 0.035 | 0.001 | | |
| **2-way ANOVA of total carotenoid content (Figure 8A)** | | | | | |
| Treatment | 1 | 0.006 | 0.006 | 3.76 | 0.06 |
| Genotype | 2 | 0.014 | 0.007 | 4.54 | **0.02** |
| Treatment x genotype | 2 | 0.032 | 0.016 | 10.31 | **<0.001** |
| Residual | 24 | 0.037 | 0.002 | | |

| **Pairwise comparisons [a]** | | **Chl *a*** | | **Chl *b*** | | **Carotenoids** | |
|---|---|---|---|---|---|---|---|
| **Fixed Factor** | **Comparison** | **q-value** | **p-value** | **q-value** | **p-value** | **q-value** | **p-value** |
| Col-0 | ctrl vs. NaCl | 7.97 | **<0.001** | 5.40 | **<0.001** | 4.84 | **<0.01** |
| *p5cs1*-4 | | 7.72 | **<0.001** | 4.38 | **<0.01** | 3.51 | **0.02** |
| *p5cs2*-1 | | 3.28 | **0.03** | 4.18 | **<0.01** | 3.60 | **0.02** |
| ctrl | Col-0 vs *p5cs1-4* | 0.80 | 0.84 | 0.88 | 0.81 | 0.60 | 0.91 |
| | Col-0 vs *p5cs2-1* | 2.06 | 0.33 | 1.90 | 0.39 | 1.22 | 0.67 |
| | *p5cs1-4* vs *p5cs2-1* | 2.86 | 0.13 | 2.78 | 0.14 | 1.82 | 0.42 |
| NaCl | Col-0 vs *p5cs1-4* | 1.04 | 0.74 | 1.90 | 0.39 | 1.94 | 0.37 |
| | Col-0 vs *p5cs2-1* | 9.18 | **<0.001** | 7.68 | **<0.001** | 7.23 | **<0.001** |
| | *p5cs1-4* vs *p5cs2-1* | 8.14 | **<0.001** | 5.78 | **<0.01** | 5.29 | **<0.01** |

[a] within fixed factors by Tukey's HSD test

**Supplementary Table S6: Statistical analyses of photosynthetic parameters in mature rosette leaves of wildtype plants and *p5cs1* or *p5cs2* mutants under non-stressed and stressed conditions.**

| Source of Variation | Degrees of freedom | Sum of squares | Mean squares | F-value | p-value |
|---|---|---|---|---|---|
| **2-way ANOVA of (ETR**/36.5)^2 (Figure 8B) | | | | | |
| Treatment | 3 | 0.311 | 0.104 | 163.40 | **<0.001** |
| Genotype | 2 | 0.049 | 0.024 | 38.45 | **<0.001** |
| Treatment x genotype | 6 | 0.049 | 0.008 | 12.99 | **<0.001** |
| Residual | 36 | 0.023 | 0.001 | | |
| | | | | | |
| **2-way ANOVA of NPQ** (Figure 8C) | | | | | |
| Treatment | 3 | 0.376 | 0.125 | 58.36 | **<0.001** |
| Genotype | 2 | 0.046 | 0.023 | 10.73 | **<0.001** |
| Treatment x genotype | 6 | 0.130 | 0.022 | 10.13 | **<0.001** |
| Residual | 36 | 0.077 | 0.002 | | |

| **Pairwise comparisons [a]** | | **ETR** | | | **NPQ** | | |
|---|---|---|---|---|---|---|---|
| **Fixed Factor** | **Comparison** | **Diff of Means** | **t-value** | **p-value** | **Diff of Means** | **t-value** | **p-value** |
| LL | Col-0 vs. *p5cs1-4* | 0.010 | 0.58 | 0.83 | -0.004 | -0.12 | 0.99 |
| | Col-0 vs. *p5cs2-1* | -0.003 | -0.17 | 0.98 | 0.048 | 1.47 | 0.32 |
| | *p5cs1-4* vs. *p5cs2-1* | -0.013 | -0.76 | 0.73 | 0.052 | 1.59 | 0.26 |
| EL | Col-0 vs. *p5cs1-4* | 0.019 | 1.06 | 0.55 | 0.008 | 0.24 | 0.97 |
| | Col-0 vs. *p5cs2-1* | -0.011 | -0.59 | 0.83 | 0.022 | 0.67 | 0.78 |
| | *p5cs1-4* vs. *p5cs2-1* | -0.029 | -1.65 | 0.24 | 0.014 | 0.43 | 0.90 |
| LL + NaCl | Col-0 vs. *p5cs1-4* | 0.016 | 0.89 | 0.65 | -0.066 | -2.02 | 0.12 |
| | Col-0 vs. *p5cs2-1* | -0.053 | -2.98 | **0.01** | -0.167 | -5.10 | **<0.001** |
| | *p5cs1-4* vs. *p5cs2-1* | -0.069 | -3.87 | **<0.01** | -0.101 | -3.08 | **0.01** |
| EL + NaCl | Col-0 vs. *p5cs1-4* | -0.005 | -0.30 | 0.95 | 0.066 | 2.03 | 0.12 |
| | Col-0 vs. *p5cs2-1* | -0.182 | -10.21 | **<0.001** | -0.164 | -4.99 | **<0.001** |
| | *p5cs1-4* vs. *p5cs2-1* | -0.176 | -9.91 | **<0.001** | -0.230 | -7.02 | **<0.001** |
| Col-0 | LL vs. EL | 0.071 | 3.99 | **<0.01** | 0.180 | 5.50 | **<0.001** |
| | LL vs. LL+NaCl | 0.089 | 5.00 | **<0.001** | 0.139 | 4.25 | **<0.001** |
| | LL vs. EL+NaCl | 0.286 | 16.06 | **<0.001** | 0.262 | 7.99 | **<0.001** |
| | EL vs. LL+NaCl | 0.018 | 1.02 | 0.74 | -0.041 | -1.25 | 0.60 |
| | EL vs. EL+NaCl | 0.215 | 12.08 | **<0.001** | 0.082 | 2.49 | 0.08 |
| | LL+NaCl vs. EL+NaCl | 0.197 | 11.06 | **<0.001** | 0.123 | 3.74 | **<0.01** |
| *p5cs1-4* | LL vs. EL | 0.079 | 4.47 | **<0.001** | 0.192 | 5.86 | **<0.001** |
| | LL vs. LL+NaCl | 0.095 | 5.31 | **<0.001** | 0.077 | 2.35 | 0.11 |
| | LL vs. EL+NaCl | 0.270 | 15.18 | **<0.001** | 0.332 | 10.14 | **<0.001** |
| | EL vs. LL+NaCl | 0.015 | 0.85 | 0.83 | -0.115 | -3.51 | **<0.01** |
| | EL vs. EL+NaCl | 0.191 | 10.71 | **<0.001** | 0.140 | 4.27 | **<0.001** |
| | LL+NaCl vs. EL+NaCl | 0.176 | 9.87 | **<0.001** | 0.255 | 7.79 | **<0.001** |
| *p5cs2-1* | LL vs. EL | 0.064 | 3.57 | **<0.01** | 0.154 | 4.70 | **<0.001** |
| | LL vs. LL+NaCl | 0.039 | 2.20 | 0.14 | -0.076 | -2.32 | 0.11 |
| | LL vs. EL+NaCl | 0.107 | 6.03 | **<0.001** | 0.050 | 1.53 | 0.43 |
| | EL vs. LL+NaCl | -0.024 | -1.37 | 0.52 | -0.230 | -7.02 | **<0.001** |
| | EL vs. EL+NaCl | 0.044 | 2.46 | 0.08 | -0.104 | -3.18 | **0.02** |
| | LL+NaCl vs. EL+NaCl | 0.068 | 3.83 | **<0.01** | 0.126 | 3.85 | **<0.01** |

[a] with general linear hypotheses

| **Source of Variation** | **Degrees of freedom** | **Sum of squares** | **Mean squares** | **F-value** | **p-value** |
|---|---|---|---|---|---|
| **2-way ANOVA of ($F_v/F_m$)**^2.8 (Figure S6A) | | | | | |
| Treatment | 3 | 1.227 | 0.409 | 193.23 | **<0.001** |
| Genotype | 2 | 0.126 | 0.063 | 29.87 | **<0.001** |
| Treatment x genotype | 6 | 0.143 | 0.024 | 11.29 | **<0.001** |
| Residual | 36 | 0.076 | 0.002 | | |
| **2-way ANOVA of** asin((**Φ PSII**)^2**)** (Figure S6B) | | | | | |
| Treatment | 3 | 0.453 | 0.151 | 147.1 | **<0.001** |
| Genotype | 2 | 0.057 | 0.029 | 28.0 | **<0.001** |
| Treatment x genotype | 6 | 0.065 | 0.011 | 10.6 | **<0.001** |
| Residual | 36 | 0.037 | 0.001 | | |
| **2-way ANOVA of** (logit(**PAR absorptivity**)^3 (Figure S6C) | | | | | |
| Treatment | 3 | 0.702 | 0.234 | 13.41 | **<0.001** |
| Genotype | 2 | 0.639 | 0.319 | 18.31 | **<0.001** |
| Treatment x genotype | 6 | 0.476 | 0.079 | 4.55 | **<0.01** |
| Residual | 36 | 0.628 | 0.017 | | |

| **Pairwise comparisons** [a] | | **$F_v/F_m$** | | | **Φ PSII** | | | **Absorptivity** | | |
|---|---|---|---|---|---|---|---|---|---|---|
| **Fixed Factor** | **Comparison** | **Diff of Means** | **t-value** | **p-value** | **Diff of Means** | **t-value** | **p-value** | **Diff of Means** | **t-value** | **p-value** |
| LL | Col-0 vs. *p5cs1-4* | 0.015 | 0.46 | 0.89 | 0.015 | 0.65 | 0.79 | -0.042 | -0.45 | 0.90 |
| | Col-0 vs. *p5cs2-1* | 0.010 | 0.30 | 0.95 | -0.004 | -0.17 | 0.98 | -0.011 | -0.11 | 0.99 |
| | *p5cs1-4* vs. *p5cs2-1* | -0.005 | -0.16 | 0.99 | -0.019 | -0.82 | 0.69 | 0.031 | 0.33 | 0.94 |
| EL | Col-0 vs. *p5cs1-4* | 0.038 | 1.16 | 0.49 | 0.022 | 0.96 | 0.61 | 0.036 | 0.39 | 0.92 |
| | Col-0 vs. *p5cs2-1* | -0.003 | -0.09 | 1.00 | -0.009 | -0.39 | 0.92 | -0.110 | -1.18 | 0.47 |
| | *p5cs1-4* vs. *p5cs2-1* | -0.040 | -1.24 | 0.44 | -0.030 | -1.34 | 0.38 | -0.147 | -1.57 | 0.27 |
| LL + NaCl | Col-0 vs. *p5cs1-4* | 0.047 | 1.44 | 0.33 | 0.015 | 0.66 | 0.79 | 0.066 | 0.71 | 0.76 |
| | Col-0 vs. *p5cs2-1* | -0.100 | -3.08 | **0.01** | -0.049 | -2.18 | 0.09 | -0.282 | -3.02 | **0.01** |
| | *p5cs1-4* vs. *p5cs2-1* | -0.147 | -4.52 | **<0.001** | -0.064 | -2.83 | **0.02** | -0.349 | -3.73 | **<0.01** |
| EL + NaCl | Col-0 vs. *p5cs1-4* | -0.018 | -0.56 | 0.84 | -0.011 | -0.49 | 0.88 | 0.167 | 1.79 | 0.19 |
| | Col-0 vs. *p5cs2-1* | -0.296 | -9.10 | **<0.001** | -0.209 | -9.24 | **<0.001** | -0.441 | -4.72 | **<0.001** |
| | *p5cs1-4* vs. *p5cs2-1* | -0.278 | -8.54 | **<0.001** | -0.198 | -8.76 | **<0.001** | -0.608 | -6.52 | **<0.001** |
| Col-0 | LL vs. EL | 0.240 | 7.38 | **<0.001** | 0.088 | 3.89 | **<0.01** | -0.035 | -0.37 | 0.98 |
| | LL vs. LL+NaCl | 0.169 | 5.19 | **<0.001** | 0.093 | 4.08 | **<0.01** | 0.300 | 3.22 | **0.01** |
| | LL vs. EL+NaCl | 0.552 | 16.98 | **<0.001** | 0.343 | 15.12 | **<0.001** | 0.258 | 2.76 | **0.04** |
| | EL vs. LL+NaCl | -0.071 | -2.19 | 0.14 | 0.004 | 0.19 | 1.00 | 0.335 | 3.59 | **<0.01** |
| | EL vs. EL+NaCl | 0.312 | 9.60 | **<0.001** | 0.254 | 11.23 | **<0.001** | 0.293 | 3.14 | **0.02** |
| | LL+NaCl vs. EL+NaCl | 0.384 | 11.79 | **<0.001** | 0.250 | 11.04 | **<0.001** | -0.043 | -0.46 | 0.97 |
| *p5cs1-4* | LL vs. EL | 0.263 | 8.07 | **<0.001** | 0.095 | 4.20 | **<0.001** | 0.043 | 0.46 | 0.97 |
| | LL vs. LL+NaCl | 0.201 | 6.16 | **<0.001** | 0.093 | 4.09 | **<0.01** | 0.408 | 4.37 | **<0.001** |
| | LL vs. EL+NaCl | 0.519 | 15.96 | **<0.001** | 0.317 | 13.98 | **<0.001** | 0.467 | 5.00 | **<0.001** |
| | EL vs. LL+NaCl | -0.062 | -1.91 | 0.24 | -0.002 | -0.11 | 1.00 | 0.365 | 3.91 | **<0.01** |
| | EL vs. EL+NaCl | 0.256 | 7.88 | **<0.001** | 0.222 | 9.79 | **<0.001** | 0.424 | 4.54 | **<0.001** |
| | LL+NaCl vs. EL+NaCl | 0.319 | 9.79 | **<0.001** | 0.224 | 9.90 | **<0.001** | 0.059 | 0.63 | 0.92 |
| *p5cs2-1* | LL vs. EL | 0.228 | 6.99 | **<0.001** | 0.083 | 3.68 | **<0.01** | -0.135 | -1.44 | 0.48 |
| | LL vs. LL+NaCl | 0.059 | 1.81 | 0.29 | 0.047 | 2.08 | 0.18 | 0.029 | 0.31 | 0.99 |
| | LL vs. EL+NaCl | 0.246 | 7.57 | **<0.001** | 0.137 | 6.05 | **<0.001** | -0.173 | -1.85 | 0.27 |
| | EL vs. LL+NaCl | -0.169 | -5.19 | **<0.001** | -0.036 | -1.60 | 0.39 | 0.163 | 1.75 | 0.31 |
| | EL vs. EL+NaCl | 0.019 | 0.58 | 0.94 | 0.054 | 2.37 | 0.10 | -0.038 | -0.41 | 0.98 |
| | LL+NaCl vs. EL+NaCl | 0.188 | 5.77 | **<0.001** | 0.090 | 3.97 | **<0.01** | -0.201 | -2.15 | 0.16 |

[a] with general linear hypotheses
