# Supplementary material for: Differential Contribution of P5CS Isoforms to Stress Tolerance in Arabidopsis
Source: Front Plant Sci. 2020 Sep 25;11:565134. doi: 10.3389/fpls.2020.565134 (PMC7545825; doi:10.3389/fpls.2020.565134)
Supplement: Supplementary file 2 [file Image_1.pdf]

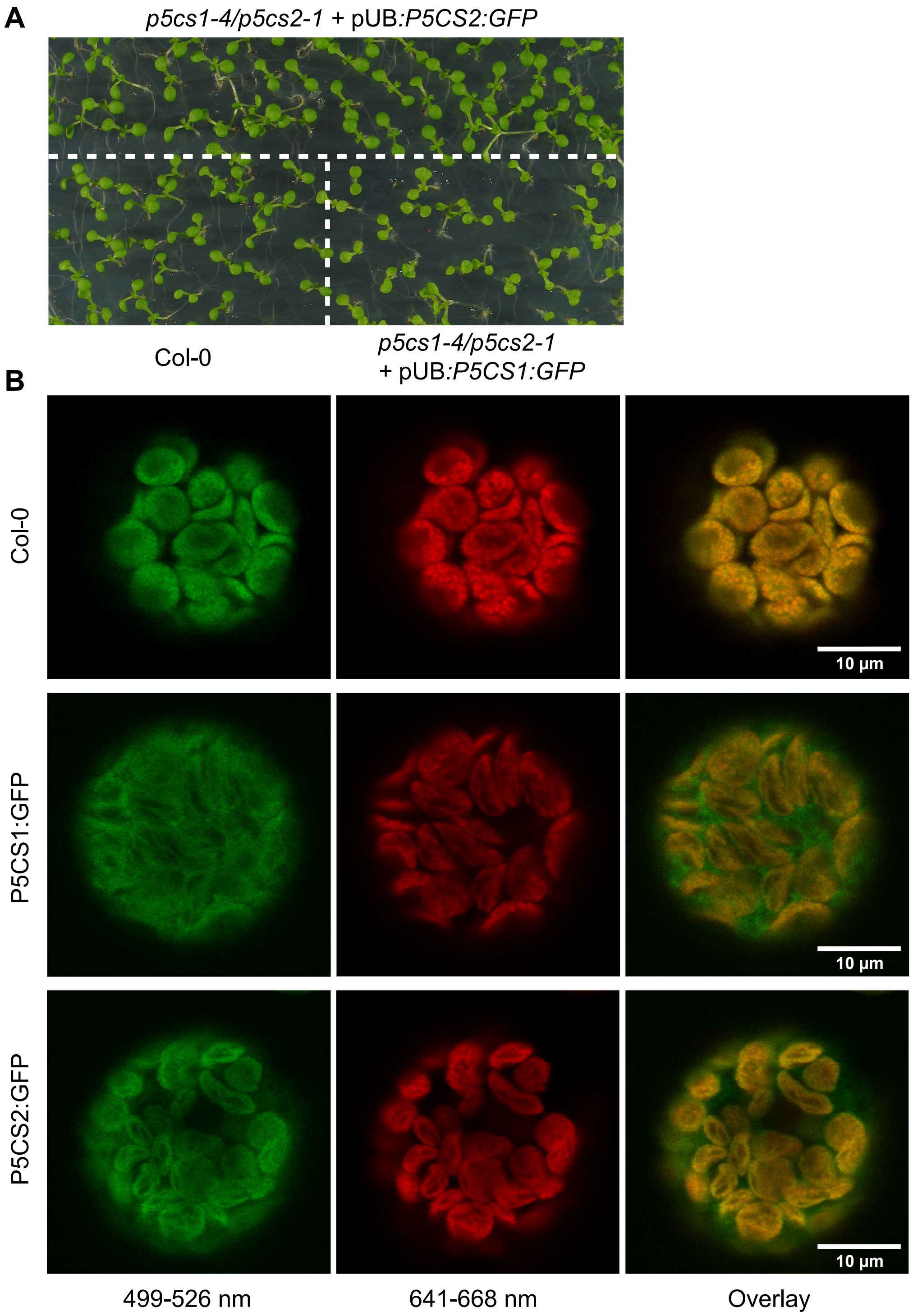

Supplementary Figure S1: Complementation of *p5cs1/p5cs2* double mutants and spectrally resolved confocal images of P5CS1:GFP and P5CS2:GFP expressing cells

(**A**) Expression of *pUB10:P5CS1:GFP* or *pUB10:P5CS2:GFP* allowed normal growth and development of *p5cs1-4/p5cs2-1* double mutants half-strength MS medium with 30 mM sucrose.

(**B**) Fluorescence images of mesophyll protoplasts isolated from wildtype, P5CS1:GFP or P5CS2:GFP expressing plants. Spectrally resolved confocal fluorescence images with 20 channels spanning 490 to 668 nm emission wavelength (the same raw data as for **Figure 1**) were used to simulate the use of GFP specific (499-526 nm emission) and chlorophyll-specific (641-668 nm emission) filters. Chlorophyll autofluorescence of wildtype protoplasts is also detected in the GFP channel and generates the impression of dual localization of P5CS1:GFP or P5CS2:GFP fusion proteins in the overlay images. (continued on next page)

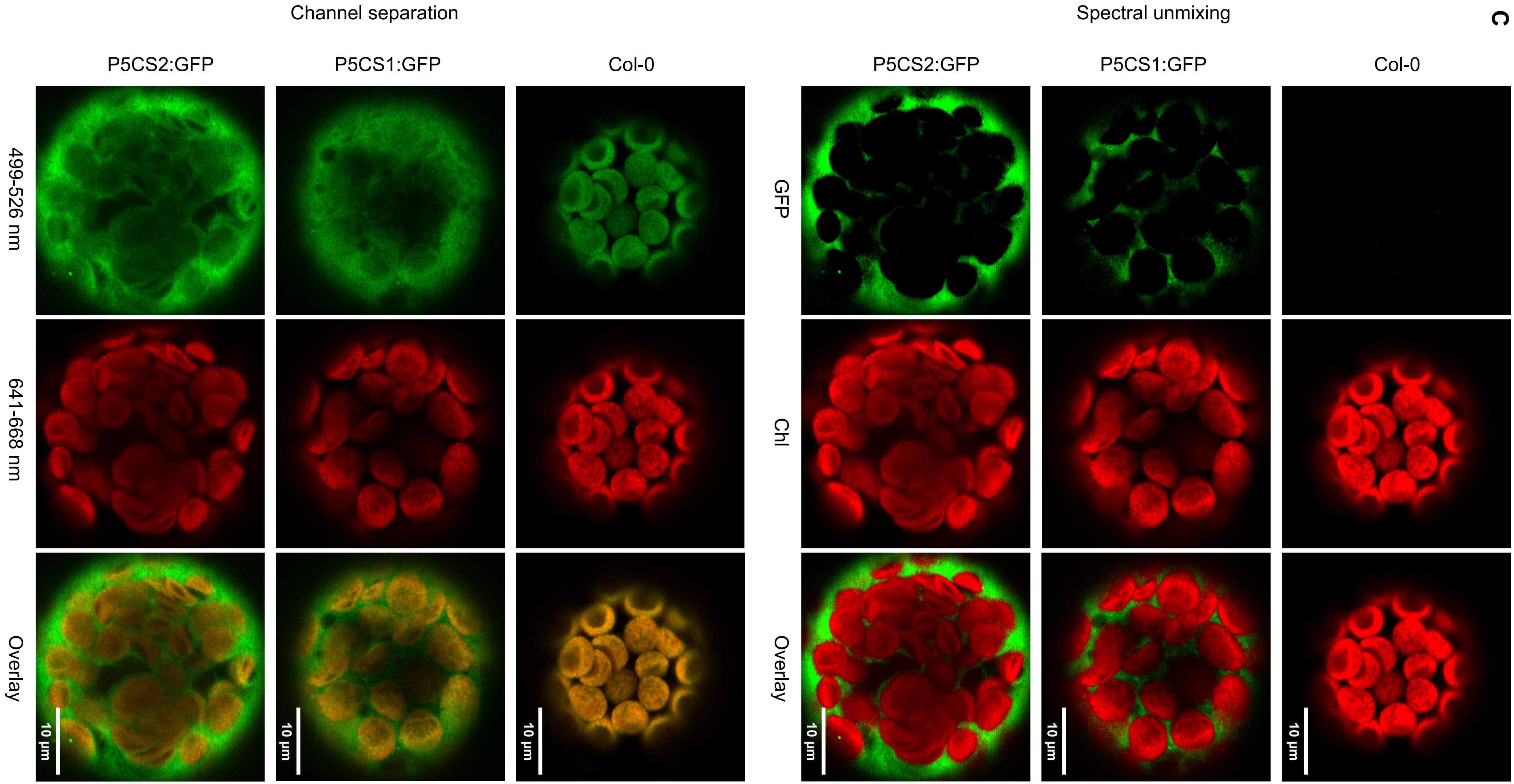

Supplementary Figure S1 (continued)**:**
(C) Confocal images of independent protoplasts to illustrate small variations in chlorophyll and GFP fluorescence intensity, that did not affect the exclusive detection of GFP fluorescence in the cytosol and chlorophyll fluorescence in chloroplasts.
