# Supplementary material for: Differential Contribution of P5CS Isoforms to Stress Tolerance in Arabidopsis
Source: Front Plant Sci. 2020 Sep 25;11:565134. doi: 10.3389/fpls.2020.565134 (PMC7545825; doi:10.3389/fpls.2020.565134)
Supplement: Supplementary file 3 [file Image_2.pdf]

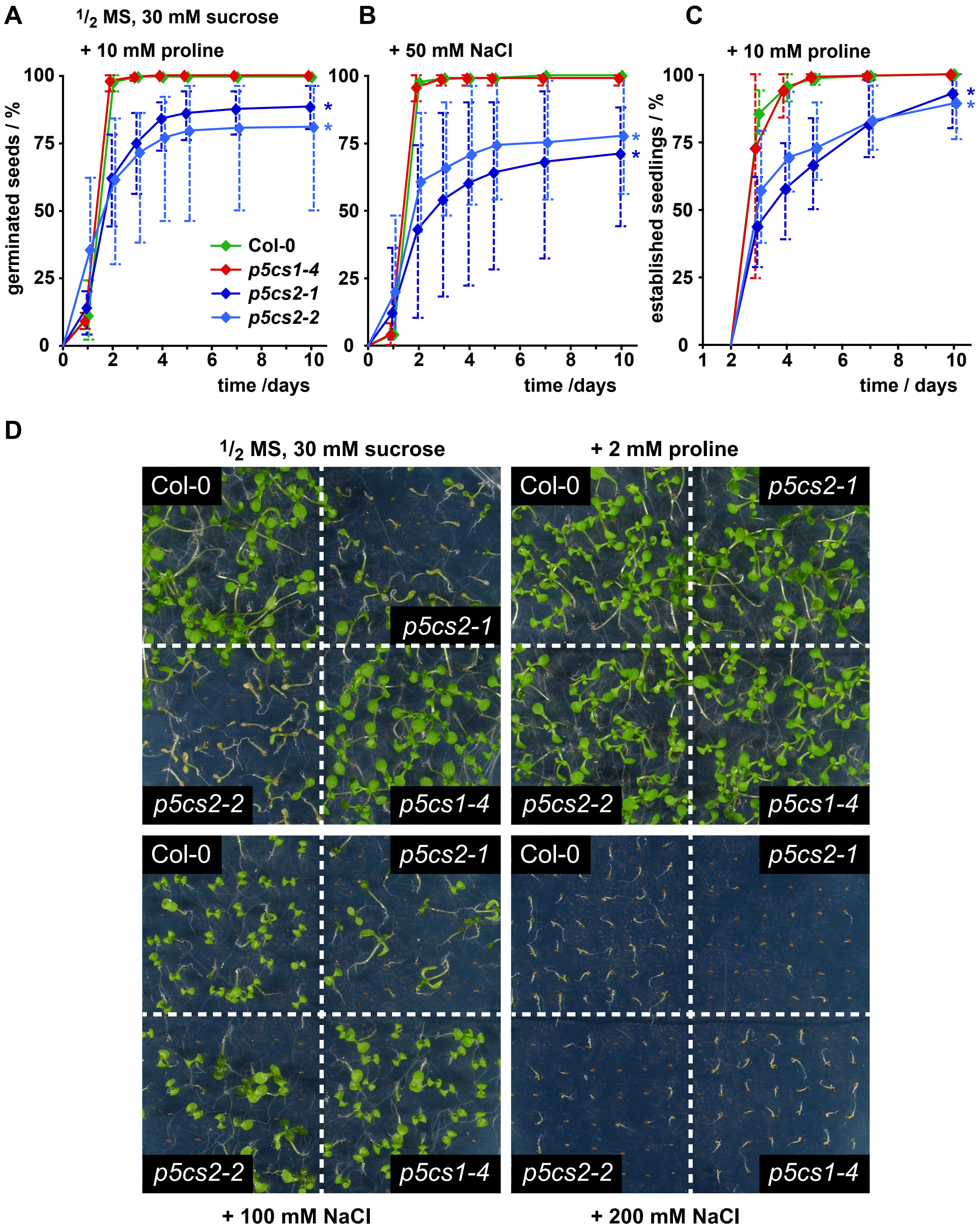

Supplementary Figure S2: Germination and establishment of *p5cs* mutants
Proportions of germinated seeds were determined between 1 and 10 days after plating of seeds on axenic half-strength MS medium containing 30 mM sucrose supplemented with either 10 mM proline (**A**) or 50 mM NaCl (**B**). Seedling establishment was determined between 2 and 10 days after plating of seeds on medium supplemented with 10 mM proline (**C**). Only seedlings with fully expanded, green cotyledons were scored as "established". Diamonds represent mean proportions of germinated seeds or established seedlings from 4 batches, consisting of 50 seeds each, from individual parental plants. Whiskers extend to the highest and lowest values in each sample and asterisks indicate values that were significantly different from Col-0 at day 10 ($p < 0.05$ by Fisher's exact test). **D**: Photographs of representative plates after 10 days under short-day conditions.
