# Supplementary material for: Differential Contribution of P5CS Isoforms to Stress Tolerance in Arabidopsis
Source: Front Plant Sci. 2020 Sep 25;11:565134. doi: 10.3389/fpls.2020.565134 (PMC7545825; doi:10.3389/fpls.2020.565134)
Supplement: Supplementary file 4 [file Image_3.pdf]

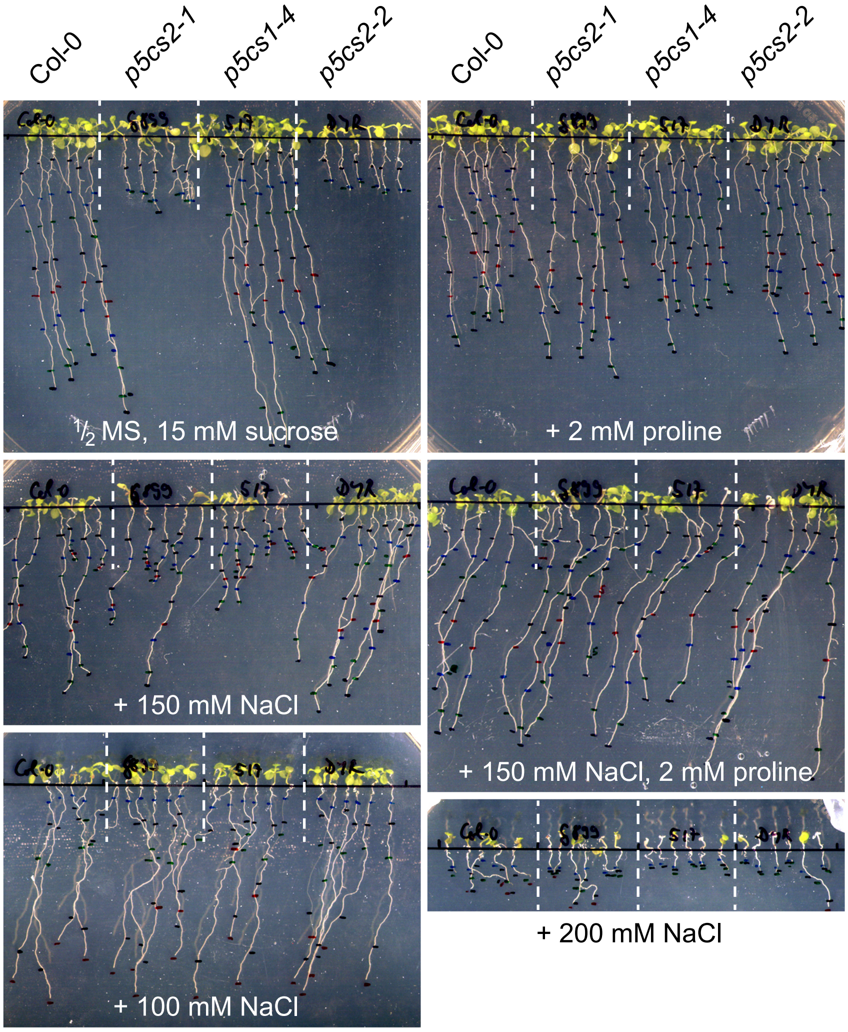

Supplementary Figure S3: Root growth of wildtype plants and *p5cs* mutants
Seedlings were cultivated for 4 days on horizontal plates with half-strength MS medium supplemented with 15 mM sucrose and 2 mM proline to support establishment of *p5cs2* mutant seedlings. Then the seedlings were transferred to vertical plates containing half-strength MS medium with 15 mM sucrose supplemented either with 2 mM proline, 100 to 200 mM NaCl, or a combination of NaCl and proline. Pictures were taken after 19 days of growth under short-day conditions.
