# Supplementary material for: Differential Contribution of P5CS Isoforms to Stress Tolerance in Arabidopsis
Source: Front Plant Sci. 2020 Sep 25;11:565134. doi: 10.3389/fpls.2020.565134 (PMC7545825; doi:10.3389/fpls.2020.565134)
Supplement: Supplementary file 5 [file Image_4.pdf]

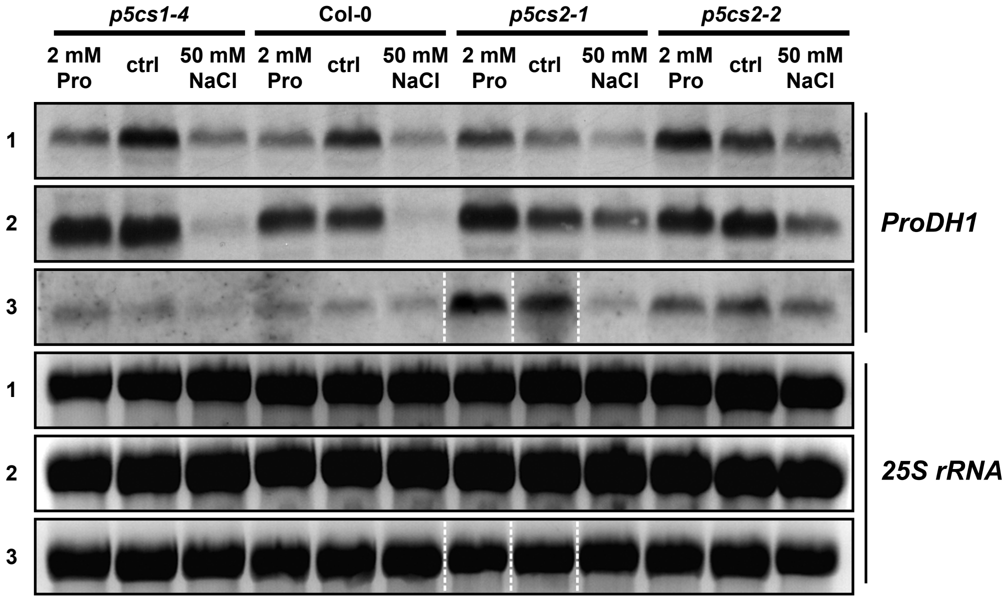

Supplementary Figure S4: High variability of *ProDH1* transcript levels

Direct comparison between the northern blot shown in **Figure 5** (lines labelled with 1) and two independent biological repetitions show that *ProDH1* transcript levels varied strongly under our cultivation conditions, but most of the genotype-specific or condition-specific differences occurred only in a single experiment. Dashed white lines indicate the digital correction of a mix-up of two samples during the loading of the RNA gel. Wildtype (Col-0) seedlings and *p5cs1* or *p5cs2* mutants were harvested after 2 weeks of growth on half-strength MS medium with 30 mM sucrose (ctrl) supplemented with 50 mM NaCl or 2 mM proline (Pro). Total RNA was extracted and subjected to northern blotting. Ethidium bromide stained *25S rRNA* is shown as a loading control in the lower three panels.
