# Supplementary material for: Differential Contribution of P5CS Isoforms to Stress Tolerance in Arabidopsis
Source: Front Plant Sci. 2020 Sep 25;11:565134. doi: 10.3389/fpls.2020.565134 (PMC7545825; doi:10.3389/fpls.2020.565134)
Supplement: Supplementary file 6 [file Image_5.pdf]

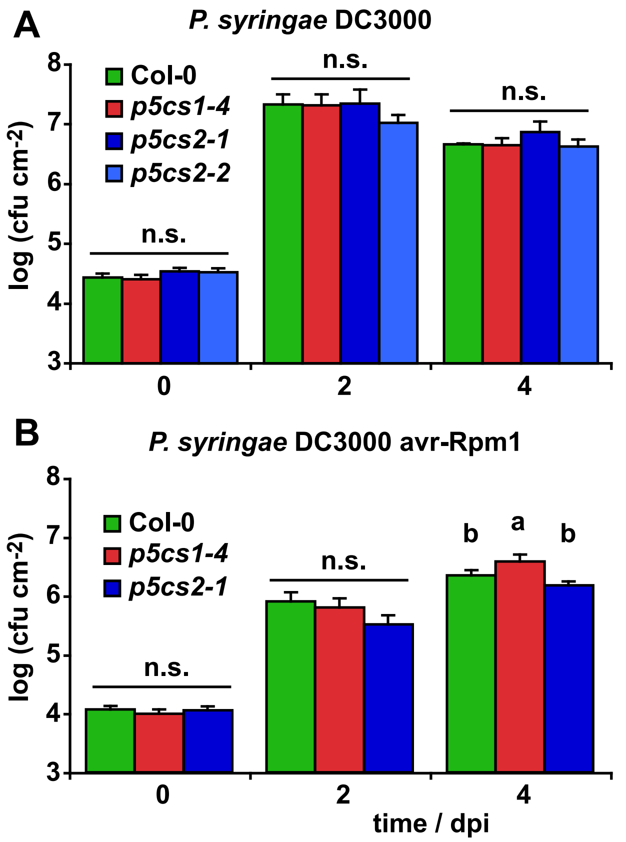

Supplementary Figure S5: Pathogen susceptibility of *p5cs1* and *p5cs2* mutants
Leaves of 5-week-old plants were infiltrated with suspensions of *Pseudomonas syringae* pv. DC3000 (A) or DC3000 avr-Rpm1, harbouring a plasmid that confers expression of the avirulence protein Rpm1 (B). Bacterial titers inside the leaves (expressed as colony forming units [cfu] per cm^2) were determined at 0, 2, and 4 days post infiltration (dpi). Columns represent the mean +SD of four samples, each pooled from three infiltrated leaves. Different letters above the columns indicate significant differences within the samples from one day ($p < 0.05$ by Tukey's HSD test, see **Supplementary Table S3**). n.s., no significant differences detected.
