# Supplementary material for: Differential Contribution of P5CS Isoforms to Stress Tolerance in Arabidopsis
Source: Front Plant Sci. 2020 Sep 25;11:565134. doi: 10.3389/fpls.2020.565134 (PMC7545825; doi:10.3389/fpls.2020.565134)
Supplement: Supplementary file 7 [file Image_6.pdf]

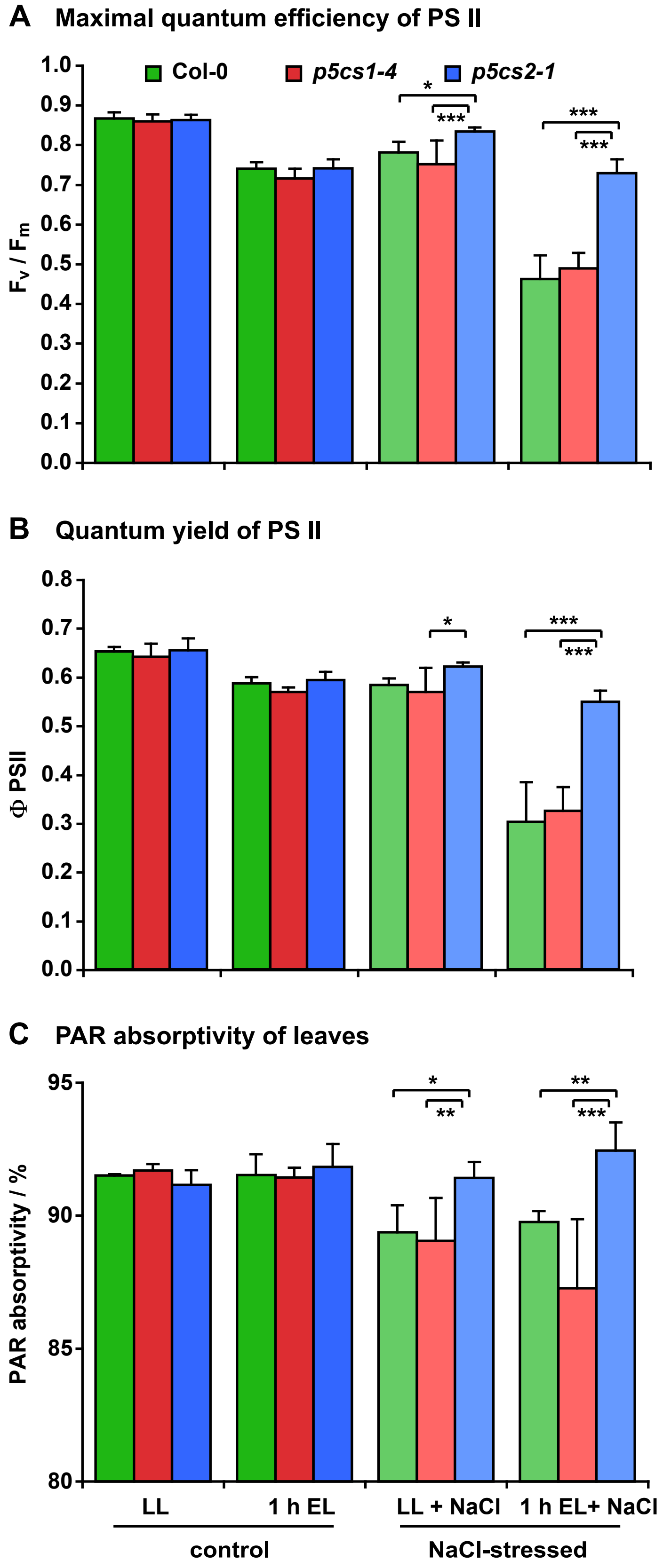

Supplementary Figure S6: Photosynthetic parameters after salt and excess light stress

Six-week-old plants were stressed with 300 mM NaCl 4 and 2 days before the analysis. The same measurements of chlorophyll fluorescence before and after 1 h exposure to excess light stress as described in Figure 8 were used to calculate the maximal quantum efficiency of PS II in the dark acclimated state (Fv/Fm; **A**), the quantum yield of PS II in the light acclimated state (ΦPSII, **B**). The absorptivity for photosynthetically active radiation (PAR absorptivity, **C**) was estimated from images of reflected red and near infrared light. Bars represent the average +SD of 4 or 5 replicates per condition and genotype. ANOVA analysis detected significant differences depending on treatment, genotype and treatment x genotype interaction in all three panels (**Supplementary table S6**). Briefly, both NaCl stress and excess light treatment reduced F_v/F_m and ΦPSII in all genotypes, but *p5cs2-1* mutants were significantly less affected. Absorptivity of photosynthetically active radiation was reduced by NaCl stress but not by excess light treatment in Col-0 and *p5cs1-4* mutants, whereas *p5cs2-1* mutants were unaffected. Asterisks indicate significant differences between genotypes within one condition (*; **; ***: p <0.5, <0.01, and <0.001, respectively).
